# Two *DRB3* residues predictively associate with the progression to type 1 diabetes among *DR3* carriers

**DOI:** 10.1172/jci.insight.184348

**Published:** 2025-03-04

**Authors:** Lue Ping Zhao, George K. Papadopoulos, Jay S. Skyler, William W. Kwok, George P. Bondinas, Antonis K. Moustakas, Ruihan Wang, Chul-Woo Pyo, Wyatt C. Nelson, Daniel E. Geraghty, Åke Lernmark

**Affiliations:** 1 Public Health Sciences Division, Fred Hutchinson Cancer Research Center, Seattle, Washington, USA.; 2 School of Public Health, University of Washington, Seattle, Washington, USA.; 3 Laboratory of Biophysics, Biochemistry, Biomaterials and Bioprocessing, Faculty of Agricultural Technology, Technological Educational Institute (TEI) of Epirus, Arta, Greece.; 4 Diabetes Research Institute and Division of Endocrinology, Diabetes & Metabolism, University of Miami Miler School of Medicine, Miami, Florida, USA.; 5 Benaroya Research Institute, Seattle, Washington, USA.; 6 Department of Food Science and Technology, Faculty of Environmental Sciences, Ionian University, Argostoli, Cephalonia, Greece.; 7 Clinical Research Division, Fred Hutchinson Cancer Research Center, Seattle, Washington, USA.; 8 Department of Clinical Sciences, Lund University CRC, Skåne University Hospital, Malmö, Sweden.

**Keywords:** Autoimmunity, Genetics, Immunology, Autoimmune diseases, Diabetes

## Abstract

*HLA-DR* genes are associated with the progression from stage 1 and stage 2 to onset of stage 3 type 1 diabetes (T1D), after accounting *HLA-DQ* genes with which they are in high linkage disequilibrium. Based on an integrated cohort of participants from 2 completed clinical trials, this investigation finds that, sharing a haplotype with the DRB1*03:01 (*DR3*) allele, DRB3*01:01:02 and *02:02:01 have respectively negative and positive associations with the progression. Furthermore, we uncovered 2 residues (β11, β26, participating in pockets 6 and 4, respectively) on the DRB3 molecule responsible for the progression among *DR3* carriers; motif RY and LF respectively delay and promote the progression (hazard ratio [HR] = 0.73 and 2.38, *P* = 0.039 and 0.017, respectively). Two anchoring pockets 6 and 4 probably bind differential autoantigenic epitopes. We further investigated the progression association with the motifs RY and LF among carriers of *DR3* and found that carriers of the motif LF have significantly faster progression than carriers of RY (HR = 1.48, *P* = 0.019 in unadjusted analysis; HR = 1.39, *P* = 0.047 in adjusted analysis), results of which provide an impetus to examine the possible role of specific DRB3-binding peptides in the progression to T1D.

## Abstract 2

Two residues in HLA-DRB3 gene have yin-yang associations with progression from stage 1 or 2 to stage 3 type 1 diabetes among carriers of DRB1*03:01.

## Graphical abstract

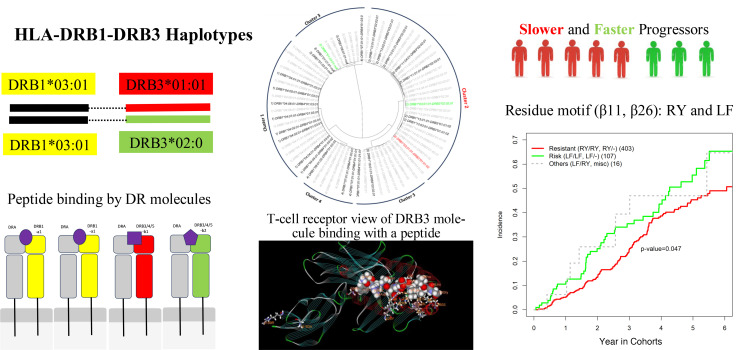

## Introduction

Type 1 diabetes (T1D) is a progressive autoimmune disease, as the host immune system recognizes, via the HLA system, autoantigenic peptides expressed by the pancreatic islet β cells, presenting the respective complexes to otherwise normal CD4^+^ T cells, initiating an autoimmune response that wrongly targets insulin-producing β cells. The cause for the initiation of such an autoimmune attack is not known, even though several hypotheses have been proposed, such as molecular mimicry to microbial antigens (1). After several years of research, 4 β cell autoantigens have been identified: insulin, glutamic acid decarboxylase 65 (GAD65), islet-associated antigen (IA-2), and zinc T8 transporter (ZnT8), highly expressed in the β cell insulin secretory granules (2, 3). Reliable standardized assays exist for all 4 of these biochemically defined autoantigens, making possible the comparison of the course of islet autoimmunity in diverse populations worldwide (2). It has, thus, become possible to follow the course of islet autoimmunity via such autoantibody assays in select populations (mostly persons at high risk because of bearing susceptible *HLA*-*II* alleles or family relationship to patients) and correlate the findings to the metabolic status of such persons (2). From such studies, it was possible to define pre-T1D stages, through months or years following the initial autoantibody formation, specific to 1 or more of these biochemically defined autoantigens; stage 1 or seroconversion is considered as the appearance of 1 or more such autoantibodies, while stage 2 or hyperglycemia reflects reduced function of the endocrine pancreas. Patients with greatly reduced numbers of β cells (approximately 80 % of all β cells) gradually develop symptomatic T1D (stage 3) (3). Hence, dissecting the genetic mechanisms underlining this disease progression from stage 1/2 disease to stage 3 T1D is essential, to develop improved secondary prevention strategies (4, 5), and is the focus of the current investigation. Following the earlier work by Butty and colleagues (6), we have integrated participants in the Diabetes Prevention Trial-Type 1 (DPT-1) and the Oral Insulin Prevention Trial (TN07) into a single cohort, referred to as iCohort, and have sequenced the *HLA* genes in all patients (6, 7). Besides replicating the earlier finding on *HLA-DQ*, our recent investigation has shown that *HLA-DR* and *HLA-DP* haplotypes appear to independently associate with progression to T1D, including the observation that *DR3* alleles (DRB1*03:01-DRB3*01:01 and DRB1*03:01-DRB3*02:02), sharing DRB1*03:01, had diametrically opposite associations with the progression (7). Furthermore, it shows that 14 residues (8 on α chain and 6 on β chain) explain *HLA-DQ* associations with the T1D progression (8), and this result expands our earlier discovery of 2 DQB1 residues (–18β, β57) that are associated with the T1D progression (9). Because of dual associations, the *HLA-DRB3* locus is likely to have an independent association with the T1D progression (7); hence, it is of interest to discover specific residues contributing to the associations, which could shed new insights into genetic mechanism governing *HLA-DR* associations with the progression.

## Results

### Genetic associations of HLA-DR (B1, B3, B4, and B5) with the progression.

When considering genetic associations of *HLA-DR*, one must consider 2 issues: a confounding effect due to high linkage disequilibrium (LD) between *DR* and *DQ*, and the presence of up to 4 *DRB* alleles in cells (Supplemental Figures 1 and 2; supplemental material available online with this article; https://doi.org/10.1172/jci.insight.184348DS1). Taking a conventional association analysis, one would assess associations with individual alleles of *DRB1* and those of *DRB3/4/5*, with or without adjusting for *HLA-DQ* genotypes (Supplemental Table 1). It appears that the adjustment for *DQ* has effectively eliminated unadjusted associations with DRB1*03:01:01 from (hazard ratio [HR] = 1.20, *P* = 0.034) in the unadjusted analysis, to (HR = 0.98, *P* = 0.95) in the adjusted analysis. Similarly, for DRB1*13:02:01, estimated resistant association (HR = 0.63, *P* = 0.023) had an insignificant association (HR = 0.075, *P* = 0.52). In the bottom panel of Supplemental Table 1, the resistant association (HR = 0.63, *P* = 0.022) with DRB3*03:01:01 becomes insignificant (HR = 0.74, *P* = 0.51). Interestingly, DRB1*01:03:01 with the relatively low frequency of 14, was shown to have a significant association in the adjusted analysis (HR = 2.46, *P* = 0.027), despite the insignificant association in the unadjusted analysis (HR = 1.68, *P* = 0.18).

Our earlier association analysis of HLA-DR with the progression considered only empirically observed haplotypes between *DRB1* and *DRB3/4/5* and have established both negative and positive associations of *DRB3*-defined *DR3* alleles (HR = 0.73 and 1.44, *P* = 0.04 and 0.02, respectively), the results from which are reproduced in Supplemental Table 2. Now accounting for 249 somatic haplotypes, by pairing *DRB1* and *DRB3/4/5* alleles (Supplemental Table 3), we grouped those rare haplotypes into a class of rare haplotypes with fewer than 10 copies and repeated the same haplotypic association analysis of DR with the progression, with or without adjusting for *HLA-DQ* (Supplemental Table 4). As expected, somatic haplotypes are more diverse than genetic haplotypes, with 39 additional common *DR* haplotypes (Supplemental Table 4), and 7 somatic haplotypes are selected, because corresponding *P* values in either unadjusted or adjusted analyses are less than 0.05 (Table 1). The first 3 haplotypes, sharing the same DRB1*03:01:01 with DRB3*01:01:02, DRB3*02:02:01, and DRB4*01:03:01, have negative, positive, and neutral associations with the progression (HR = 0.73, 1.35, and 0.80; *P* = 0.01, 0.02, and 0.53, respectively), in which DRB1*03:01:01- DRB4*01:03:01 (h, indicating that they are not empirically observed in the population) is a uniquely somatic haplotype. The variable associations, while sharing the same DRB1*03:01:01, support that DRB3*01:01:02 and DRB3*02:02:01, but not DRB4*01:03:01, likely play a functional role.

Next, 2 genetic haplotypes (DRB1*01:01:01-null and DRB1*01:01:01- DRB3*01:01:02) share the same DRB1*01:01:01 but have negative and positive associations with the progression in the adjusted analyses (HR = 0.35 and 2.14, *P* = 0.005 and 0.03, respectively). Finally, DRB1*07:01:01 alone, as a somatic haplotype, has a persistently positive association with the progression in both unadjusted and adjusted analyses (HR = 2.04 and 2.85, *P* = 0.04 and 0.005, respectively), while other DRB1*07:01:01 haplotypes have no significant associations. Meanwhile, the association of DRB1*13:02:01-DRB3*03:01:01 observed in the unadjusted association analysis seems to be accounted for by *HLA-DQ*.

### Amino acids (β11 and β26) in DRB3 are responsible for associations with the progression.

**Figure 1:**
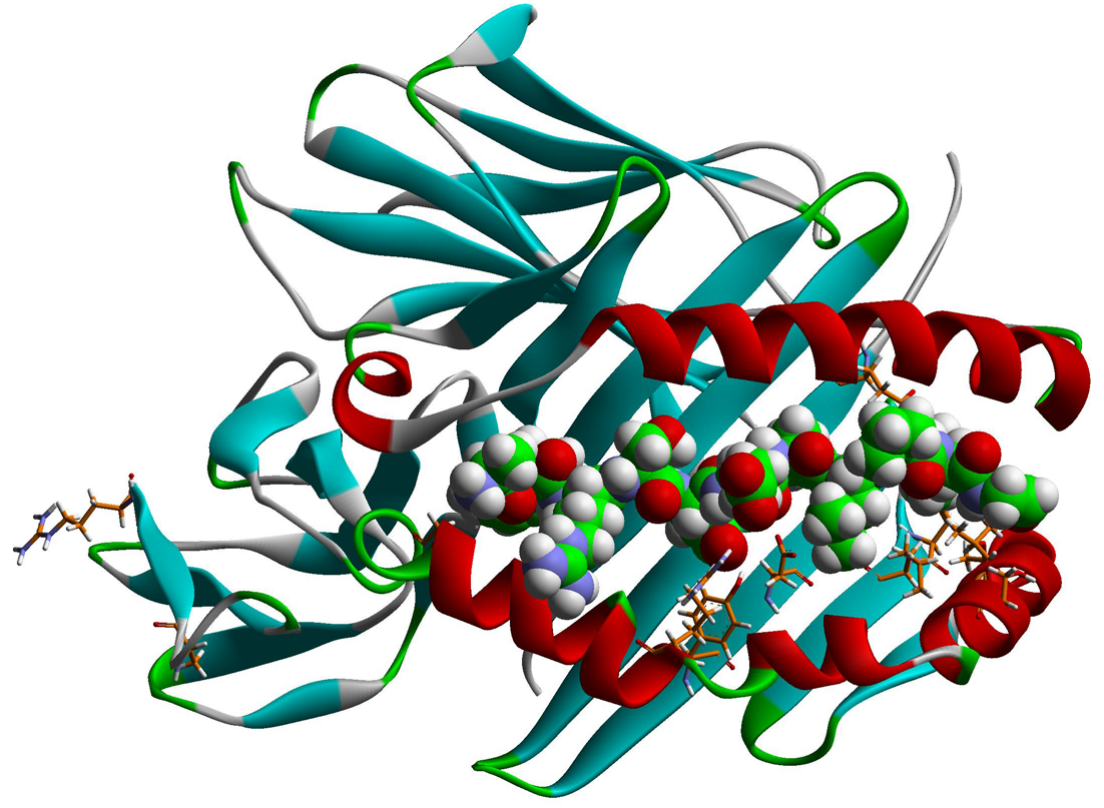
Tilted T Cell Receptor view of HLA-DRB3*01:01 in complex with the platelet integrin peptide. Tilted T Cell Receptor view of HLA-DRB3*01:01 in complex with the platelet integrin peptide associated with fetal and neonatal alloimmune thrombocytopenia (peptide sequence AWRSDEALPLG, anchors in bold, 2Q6W.pdb) (15). Tilting was done so that as many of the involved residues would be exposed to view. The concerned residues are β11, β26, and β86, as shown by HOH analysis, and residues β28, β30, β37, β38, β51, β57, β60, β74, β183, and b189, which are in absolute LD to β26 in all DRB3 alleles. The secondary structure of the 2 polypeptide chains of DRB3 (α and β) are shown as line ribbons, colored according to their different secondary structural elements: α-helix in red, β-sheet in turquoise, and β-turn and random coil in green. The residues of the antigenic peptide are in space-filling form (atomic color convention: carbon, gray; oxygen, red; nitrogen, cyan; hydrogen, white; sulfur, yellow) and labeled in 3-letter code. The pertinent residues shown to be significant in HOH analysis, and those in absolute LD with β26, are in stick form, with the same color convention.

While sharing haplotypes with *DR3*, different *DRB3* alleles evidently have both positive and negative associations with the progression. Extracting *DR3*-shared haplotypes from the haplotype association analysis (Supplemental Table 3), Table 2 lists 4 genetic haplotypes with *DRB3* alleles, including 2 rare haplotypes (DR3-DRB3*01:02 and DR3-DRB3*02:24). Also extracted are 3 polymorphic amino acids across these 4 haplotypes, which include 3 residues (β11, β26, β86). Since residue β86 is polymorphic across the rare DRB3*02:24 allele, only the first 2 (β11, β26) are polymorphic between DRB3*01:01:02 and DRB3*02:02:01 with 2 distinct motifs RY and LF. Residue β11 is an integral part of pocket 6 of the antigen-binding groove of HLA-DR/DQ molecules; likewise, β26 is an integral part of pocket 4 of the same group of molecules (10–12). Note that β26 is in complete LD with 10 other residues (β28, β30, β37, β38, β51, β57, β60, β74, β183, and β189), and the corresponding motifs are YDYFLTVSRAR and FEHYARDYQPS, in which amino acid β26 is underscored. It so happens that these residues concern pockets 4 (β74), 6 (β30), 7 (β28), and 9 (β37, β38, and β57) in the β1 domain (residues 1–90), while β51 is part of the β49–β56 homodimerization patch of HLA-DR molecules (12, 13) (Figure 1 and Supplemental Figure 1). The last 2 residues (β183 and β189) are very close to residues β184Leu and β187Glu that come in contact with HLA-DM during the peptide exchange between HLA-II–bound Class II-associated Invariant Chain Peptide (CLIP) and higher-affinity antigenic peptides, taking place in the endosome (pH 5.5) (10, 14, 15) (Supplemental Figures 2 and 3). In particular, DRβ189Arg interacts with β187Glu so that the latter remains fixed to interact with HLA-DMβ110Arg. It cannot be easily surmised what the effect on this interaction would be by the β189Arg→Ser substitution — i.e., stronger DRβ187Glu-DMβ110Arg interaction or a weaker one, meaning a stronger DM-DR interaction — as both charged residues are free to assume different rotamer orientations (Supplemental Table 5). This interaction is complemented by the interaction of DMβ108Asn with DRβ96Glu, the weak cation-π interaction of DRβ98Lys with DMβ107Phe, and the several weak hydrophobic interactions among DRβ residues 99Val, 100Thr, 101Val, 184Leu, 185Thr, and 186Val (15, 16). However, in all DRB3 allelic molecules implicated in T1D in our analyses, residue β96 is a His instead of a Glu, and β98 in all but 1 is a Gln instead of a Lys (Supplemental Figure 2), probably weakening these interactions.

### High-risk DR3-DRB3 motif association with faster progression.

**Figure 2:**
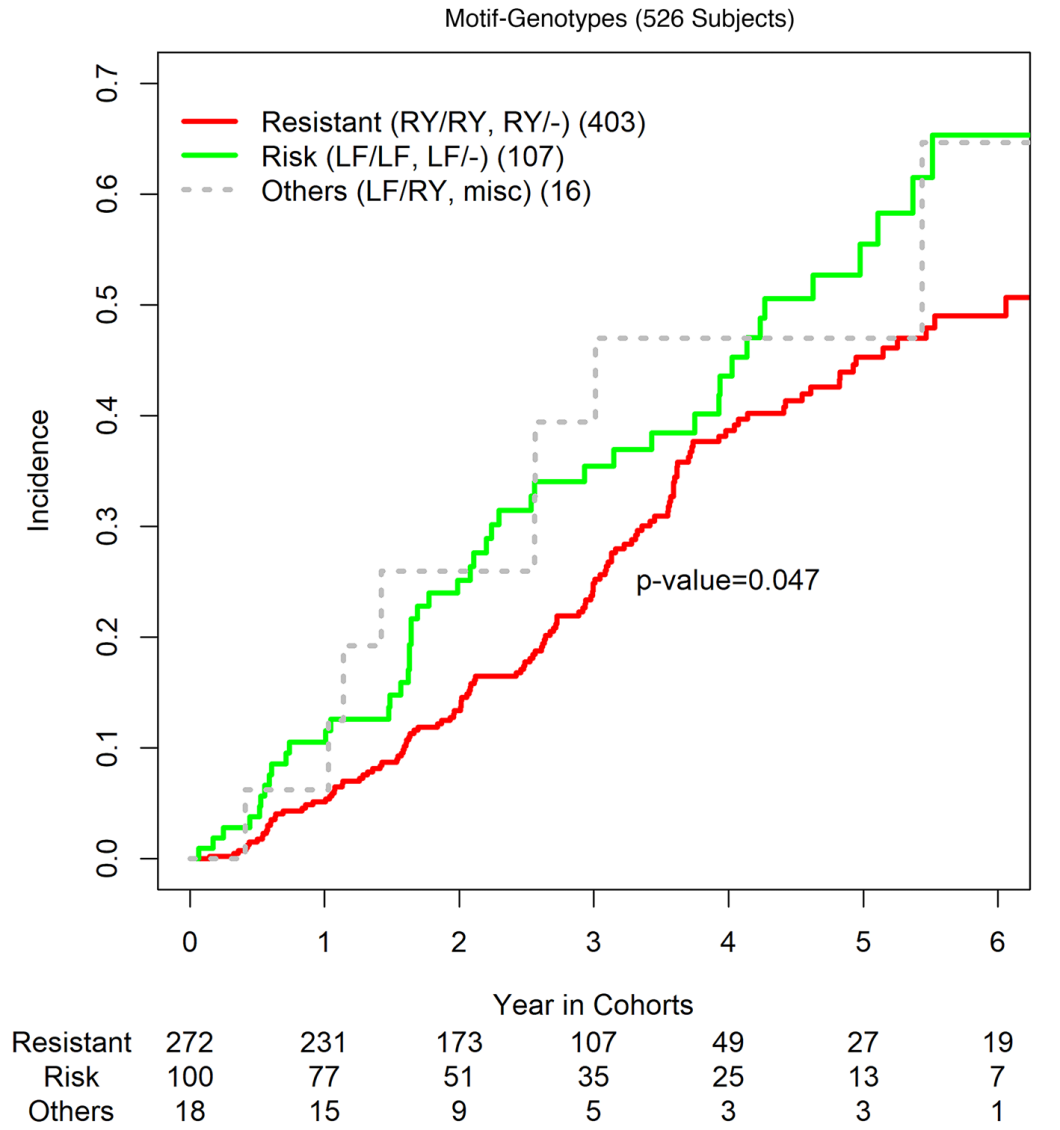
Incidence curves by motif-genotype groups. Slower progressors (Resistant, RY/RY, RY/–), faster progressors (Risk), and others.

To gain insight of genotypic associations of motifs (β11, β26), we restrict the investigation to 526 carriers of *DR3-DQ2.5* so that *HLA-DQ* genotypes are effectively adjusted. To account for those non-*DR3* haplotypes, coupling with *DR3-DQ2.5*, we haplotyped *DRB1-DRB3/4/5* and *DQA1-DQB1* and established a linkage of *DR-DQ* haplotypes with the motifs β11 and β26 (Supplemental Table 4), in which haplotypes are sorted by motifs and *DR-DQ* haplotypes and in which haplotypes of LF, RY, and AN are highlighted in green, red and gray, respectively. In total, there are 15% haplotypes that carry motif LF, 43% carry motif RY, and 37% carry motif AN together rare motifs CY, DF, or missing motifs. The “–” symbol includes all motifs other than LF and RY, and genotypic associations of motifs RY and LF with genotypic frequencies can be evaludated as follows: 1 LF/LF, 13 LF/RY, 106 LF/–, 30 RY/RY, 373 RY/– and 3 –/–. These motif genotypes are then grouped into resistant group (RY/RY, RY/–), risk group (LF/LF, LF/–), and others (LF/RY, –/–). Taking the resistant group as a reference, participants in the risk group are found to have significantly accelerated progression toward T1D onset (HR = 1.48, *P* = 0.019) (Table 3). After adjusting for risk level, age, and study cohort, this association remains to be significant (HR = 1.39, *P* = 0.047), which is consistently supported by their incidence curves (Figure 2). Note that age at the baseline (in decade) is found to inversely associate with the progression (HR = 0.54, *P* = 1.16 × 10^–6^).

### Structural properties of β11, β26 in DRB3.

The 2 residues primarily shown to relate to progression to T1D as well as resistance to it, in persons participating in either 1 of the 2 oral insulin clinical trials, are important components of pocket 6 (β11) and pocket 4 (β26) (Figure 1 and Supplemental Figures 2 and 3). The dimorphisms in question for the DRB3 molecules correlating with resistance/progression to T1D among the participants in the trials are β11Arg/Leu and β26Tyr/Phe, respectively. In both cases, residues have distinctly different physicochemical properties that, by extension, affect different anchor preferences in the respective pockets and, hence, different autoantigenic epitopes for binding to the respective DRB3 molecule. It is of even greater interest that several polymorphic DRB3 residues are in absolute LD with the β26 residue; they are involved in various functions of the HLA-DRB3 molecule, from antigen binding to cognate TCR-induced homodimerization, cognate TCR contact/interactions, and possibly HLA-DM–mediated endosomal CLIP-antigenic peptide exchange (Supplemental Figures 2 and 3) (10, 13, 14).

## Discussion

The current investigation is a post hoc study of T1D onset outcomes collected from participants in 2 randomized clinical trials, with newly sequenced *HLA* genotypes, and the results are informative but exploratory. Even though the results are less definitive than those to be expected from clinical trials with predefined and specific hypotheses, obtained association statistics and *P* values are suggestive, especially in the context of searching for specific responsible amino acids for DR associations with the progression to T1D. By the prespecified threshold of significance level at 5% without correcting for multiple comparisons, we have identified 2 residues (β11, β26) in DRB3 molecules, and their motifs (RY, LF), among *DR3-DQ2* carriers, are significantly associated with the progression from stage 1/2 disease to stage 3 T1D onset (HR = 0.73 and 1.45, *P* = 0.039 and 0.017, respectively). When comparing the group of risk genotypes (LF/LF, LF/–) with the group of resistant genotypes (RY/RY, RY/–), participants in the former group have significantly accelerated progression (HR = 1.48, *P* = 0.019). The incidence curves in both groups show that participants with high-risk motif genotypes have faster progressions throughout follow-up periods (Figure 2).

### Possible DR3-DRB3 interactions.

Both positive and negative associations of *DRB3*, exclusively among those with DR3*^+^* haplotypes, can be conceptualized as *DR3-DRB3* gene and/or protein interactions, since *DR3* and 2 *DRB3* alleles are not marginally associated with the T1D progression after adjusting *HLA-DQ* (Supplemental Table 1). While *DR3* shared haplotypes only with *DRB3* alleles, the latter share with other *DRB1* alleles, but none of them have associations with progression to T1D. The *DRA-DRB3* complexes may recognize autoantigenic peptides and have positive correlation with such progression to T1D. Empirically, such an association may appear as DR3-DRB3 interactions, or yin-yang associations. Thus far, there have been no recorded T1D autoantigenic epitopes restricted to any DRB3 molecules (17). A careful inspection of the DRB3 amino acid sequences in the antigen-binding domain reveals distinct differences in pockets 1, 4, 6, and 9 between DR3 and any of the said DRB3 molecules, such that any 2 could hardly bind with similar strength to the same autoantigenic epitope (Supplemental Figure 3). To our knowledge, there have been very few studies on the regulation of expression on the APC surface of the DRB3/4/5 αβ protein complexes, in comparison with the respective DRB1 αβ complexes. For example, monoclonal antibody 7.3.19.1, specific for epitope 77N, recognizes all DRB3 alleles except DRB3*03, and has been used to assess the membrane protein expression of this gene locus in transplanted kidneys where there was *HLA-DRB3* mismatch and consequent expression of anti-DRB3–specific antibodies to the allelic protein found in the donor but not in the recipient. The expression of DRB3 protein at the membrane of CD19^+^ B lymphocytes was about the same, regardless of the specific allele, even though there was considerable allelic variation at the level of mRNA expression in the same cells (18). In other studies, it was shown that the cell membrane expression of the DR3 protein was about 3 times that of its DRB3 counterpart, while in general, the abundance in mRNA levels was also higher for DRB1, yet there were individuals in which the ratio of DRB1/DRB3 protein was less than 2 (19, 20). It is of interest, nonetheless, that the signal peptide of the DRB1*03:01 protein is quite different from its DRB3 allelic protein counterpart (all but very few of DRB3 molecules with known sequences have identical signal peptides); both DRB1*03:01 and DRB3 signal peptides are different in 3 and 5 of 30 residues, respectively, compared with their counterpart in DR1 (Supplemental Figure 2); in the case of the DRB3 signal peptide, 3 of the substitutions are completely nonconservative. The study of the regulation of *HLA-DRB1/DRB3* expression certainly must examine such outstanding questions, especially as it concerns the biosynthesis and appearance on the cell membrane of APCs of functional HLA class II molecules (21). It is particularly intriguing that new single nucleotide polymorphisms (SNPs) and enhancers are discovered within the human *HLA-II* locus, often having a direct connection to given autoimmune diseases, including T1D (22, 23). Therefore, studying the in vivo conditions for upregulation of membrane expression of HLA-II proteins on APCs is of utmost importance, as the density of these proteins in complex with given epitopes is what determines the specific autoimmune response (24). In this regard, the study of the expression of HLA-II proteins by human islet macrophages in close proximity to human islet β cells is of paramount importance to understanding T1D pathogenesis (25).

### Structural insights into β11 and β26 (plus residues in absolute LD with the latter).

Residues β11 and β26, shown to be distinct between the allelic molecules susceptible and resistant to T1D progression, are in pockets 6 and 4, respectively. In the context of the DRB3 alleles examined in this study, 10 other DRB3 residues are in complete LD with β26, of which 6 constitute parts of pockets 4, 6, 7, and 9, with one of them being the pivotal β57 residue that determines the nature of the p9 anchor residue as well as the stability of the pMHCII complex and the sequence of the cognate TCR (10, 11, 13, 26, 27). Thus, these 2 DRB3 alleles should bind to different antigenic peptides. Indeed, it has been demonstrated that DRB1*03:01, DRB3*01:01, and DRB3*03:02 present different antigenic epitopes (20). DRB1*03:01 has the rare property of p9-anchor preferences dependent on p6 anchors because of its b11Ser/b9Glu combination (28); DRB3 allelic molecules might not share this property. DRB3 autoantigenic peptides derived from the 4 biochemically defined autoantigens of T1D (3, 10, 29) have not yet been identified. The lack of any DRB3-restricted CD4 T cells (effector or regulatory) specific for any such T1D-linked autoantigenic epitope cannot allow us to speculate any further (17). It is also interesting that 2 of those 10 residues that are in absolute LD with β26 are in the DRβ2 interaction surface with HLA-DMβ2 during the DM-mediated HLA-DR–CLIP/antigenic peptide exchange that takes place in the endosome at pH 5.5 (14, 30) (Supplemental Figures 2 and 3). The latter property is especially intriguing, as the weak exchange of CLIP with antigenic peptide happens to be a property of both HLA-DQ2 and HLA-DQ8 molecules that both confer susceptibility to T1D and to celiac disease (30, 31). In fact, the weak ability of HLA-DM to exchange CLIP with antigenic peptides for these 2 HLA-DQ molecules in the mildly acidic environment of the endosome has been considered as a contributing factor to T1D pathogenesis (30, 31). Our results do not suggest specific autoantigenic epitopes responsible for the observed effects, yet an investigation for putative autoantigenic peptides would probably start with GAD65, since autoantibodies to it are linked to DR3-DQ2 (32), and should record both effector as well as CD4 Tregs so restricted and specific to GAD65 and/or other autoantigens (33–35). We believe that an experimental investigation of the ability of the specific DRB3 molecules, one susceptible and another resistant to T1D, to exchange CLIP with other antigenic peptides is also necessary to clarify this point, as part of a general search into specific CD4^+^ T cell sensitization restricted to these DRB3 molecules (36, 37).

### Possible confounding from HLA-DP and HLA class I genes.

Given the profound role of *HLA-DQ* and tight LD, our investigation has carefully addressed *HLA-DR* associations with or without adjusting for *HLA-DQ* as possible confounding effects. One possible limitation is that *HLA-DP* may have some confounding effects, due to its independent contribution to the progression (7) and its LD with *HLA-DR*. Similarly, *HLA class I* genes (*A, B, C*) may also have some confounding effects, due to their LD with *HLA-DR* and possible associations with the progression. However, LD of *DR* with *DP* is modest and that with *HLA class I* genes is even weaker, so that their confounding effects are expected to be minimal. Nevertheless, such effects should be further investigated.

## Methods

### Sex as a biological variable.

Both male and female participants in DPT-1 and TN07 were included in this investigation (Supplemental Table 6). Since sex was not significantly associated with the progression, it was not considered as a biological variable in the genetic analysis.

### Diabetes prevention trials (DPT-1 and TN07).

DPT-1 and TN07 were randomized clinical trials to assess efficacy of oral/parenteral insulin for preventing T1D or slowing disease progression, which have been detailed elsewhere (6, 7, 38). Briefly, DPT-1 and TN07 recruited, respectively, 670 and 546 participants who were at stages 1 or 2 of disease and were integrated into a so-called integrated cohort (iCohort), totaling 1,216 participants (Supplemental Table 5). Participants’ ages were negatively associated with the progression risk, while high risk levels at baseline associated with the faster progression. For the current investigation, both are included as possible confounding factors to be adjusted throughout, unless noted otherwise.

### Genotyping HLA using next-generation targeted sequencing (NGTS) technology.

*HLA* typing was carried out using the Scisco HLA v6 typing kit (Scisco Genetics Inc.) following the kit protocol. Briefly, the method employs an amplicon-based 2-stage PCR, followed by sample pooling and sequencing using a MiSeq v2 PE500 (Illumina). The protocol yielded 3-field coverage of *HLA-II* genes (*DRB1*, *DRB3*, *DRB4*, *DRB5*, *DQA1*, *DQB1*, *DPA1*, and *DPB1*). Phase within each gene was determined in part by bridging amplicons and, whenever not available, by database lookup *HLAII* (39). *HLA-DQ* haplotypes are inferred from *DQA1* and *DQB1* genotypes, owning to their strong LD (7). Similarly, we infer the *HLA-DR* haplotype between *DRB1* and *DRB3/4/5*, since at most 1 of *DRB3*, *DRB4*, or *DRB5* linked with *DRB1* and they are in high LD. Inferred DR haplotypes are empirically observed and are referred to as genetic haplotypes. In contrast, 2 DRB1 alleles can somatically be paired with 2 DRB3/4/5 alleles (Supplemental Figure 1), forming up to 4 distinct DR haplotypes. Some of somatically generated DR haplotypes are not observed among genetic haplotypes and are thus referred to as “somatic haplotypes.”

Two samples are not genotyped for DR genes due to limited DNA. Out of 1,214 *DR* genotypes, 96.2% of samples have unambiguously inferred *DR* haplotypes (100%). Among the rest, 31 samples (2.5%) have inference posterior probability greater than 0.99, 8 (0.66%) are with posterior probability greater than 0.95, and 7 (0.68%) are with posterior probability between 0.71 and 0.84. With minimum effect from small haplotype construction variations, all analyses use *DR* haplotypes with the highest posterior probability as inferred haplotypes.

In order to maintain structural equivalence in HLA-DQ protein residues, we adopted the numbering system of Fremont et al. based on mouse H2–A (29) and modified by Bondinas et al. for MHC II molecules (12). In this manner, essentially all residues that occupy identical or near-identical positions in the respective structures have the same residue numbers in the sequence, taking into account all possible insertions and deletions in certain alleles. In the case of *HLA-DRB3* numbering of the *HLA-DR* AA sequence releases, we note that the 2020 release showed an insertion between β25 and β26 in the mature sequence, albeit without altering the standard numbering sequence (https://raw.githubusercontent.com/ANHIG/IMGTHLA/Latest/alignments/DRB_prot.txt). By contrast, the 2024 release did not show such an insertion but, instead, showed a 3-residue insertion between β66 and β67, albeit without any change in the standard numbering sequence (Supplemental Figure 2). This insertion was not present in any of the HLA-DRB3 alleles found in the subjects of the study. AA abbreviations are mostly in the 1-letter convention and occasionally in the 3-letter convention.

Within the iCohort, a total of 73 *DR* genetic haplotypes were empirically observed (Supplemental Table 7), and their associations were reported earlier (7). Considering all possible pairs of *DRB1* and *DRB3/4/5* alleles, we found that there are 249 DR somatic haplotypes (Supplemental Table 3), in which 63 DR somatic haplotypes (heterodimers) have more than 10 copies (Supplemental Table 8). Since there are 2 monomorphic *DRA* alleles available to form a DR molecule, it is expected that 2 of up to 4 *DRB1/3/4/5* are “randomly” paired with DRA to form the αβ assembly of the DR molecule (Supplemental Figure 1).

To gain insights into DR haplotypes, we have used unsupervised learning technique to organize all haplotypes in a hierarchical manner. Specifically, for 73 genetic haplotypes (Supplemental Table 7), we computed genetic distances of allele-specific amino acids between all haplotypes, and we used hierarchical clustering to organize all DR haplotypes and to display them in a form of a hierarchical tree (Supplemental Figure 4). Similarly, for 249 heterodimers, we applied the same procedure to organize and display all DR heterodimers (Supplemental Figure 5).

### Molecular depictions.

Graphical rendering of the HLA-DRB3*01:01 molecule in complex with an integrin peptide and the HLA-DR1—HLA-DM complex was performed using the respective crystal structures as deposited in the Protein Data Bank and detailed in the legends of the respective figures. We use the DSViewerPro (v. 6) rendering program of Accelrys. Details of the rendering conventions are listed in the legends of the individual figures.

### Statistics.

Given the analytic objective, statistical approaches used in this investigation were developed for analyzing polymorphic *HLA-II* genes and HOH association analysis, which have been detailed elsewhere (7, 8). Briefly, our investigation is most appropriately considered as a post hoc analysis and is exploratory in nature with computed *P* values to quantify magnitudes of observed associations, even though both DPT-1 and TN01 are randomized clinical trials. Second, this investigation is based on the observation that *HLA-II* genes (*DR*, *DP*, *DQ*) are independently contributing to the disease progression, and the analytic objective is to uncover which AAs in HLA-DR are responsible for observed DR associations — i.e., exploring specific “association signals” in the “alternative domain,” rather than a “fishing expedition” in an “open sea.” By adhering to a strict interpretation of *P* values, one could apply multiple comparison corrections — e.g., classical corrections (40) or false discovery rates (41) — but such corrections may have with multiple genes, multiple alleles, and multiple adjustments to account for, which lead to excessively conservative and variable *P* value corrections without explicitly addressing highly polymorphic *HLA-II* genes and alternative domains with strong associations with a few alleles. We thus choose to use uncorrected *P* values and a threshold of *P* = 0.05 to identify “significant associations,” while disclosing all computed *P* values to minimize “selection biases.” For readers with a particular interest, they could formulate their own specific corrections as appropriate. Third, “negative associations” could be associated with small numbers of observations, which, not surprisingly, are common for *HLA-II* gene alleles/AA and need to be interpreted cautiously. Fourth, to enable a regression analysis with genetic haplotypes, we transformed DR haplotype pair 
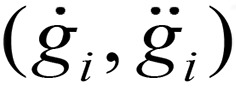
 on each *i*^th^ individual to a vector of haplotype counts, 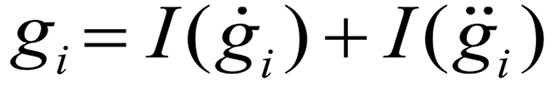 , in which 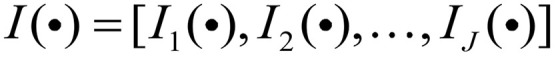 is a vector of indicators for *J* unique haplotypes and 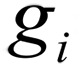
takes a value of 0, 1, or 2 corresponding to number of a specific haplotype. Similarly, for a somatic regression analysis, we transform the haplotype pair 
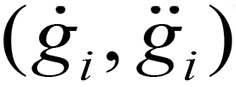
 to a vector of somatic haplotype counts, 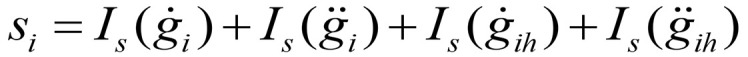, in which the suffix “*h*” denotes trans-haplotypes, 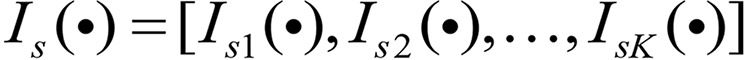 is a vector of indicators for *K* unique somatic haplotypes, and 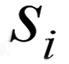 takes a value of 0, 1, 2, 3, or 4, corresponding to the number of different alleles in a specific somatic haplotype. Fifth, *HLA-II* genes, unlike typical SNPs, are highly polymorphic and have many alleles with relatively modest frequencies — e.g., 10 or more copies in the current study. In fact, some of these alleles, despite modest frequencies, could be functionally important. However, due to relatively modest frequencies, powers to resolve their associations are relatively low; consequently, the corresponding *P* values could be larger than 0.05. Hence, in the current context, we need to be cautiously calling an allele as neutral with *P* value greater than 0.05, when the associated frequencies are very small. Lastly, on statistical tools, our investigation relies on statistical functions and packages in R. For haplotyping *DRB1* and *DRB3/4/5*, we use the “haplo.em” function from the R package “haplo.stats.” For assessing genetic associations with the time-to-onset of T1D, we use the “coxph” function, with appropriate adjustment for confounding variables and *HLA-DQ* alleles.

### Study approval.

This study used archived data and anonymized DNA samples and was approved as “exemption” by the IRB at Fred Hutchinson Cancer Center (IRB no. RG1001816). Furthermore, the application to access to clinical data from resources from NIDDK Central Repository was reviewed and approved in accordance with the data access policies.

### Data availability.

All underlying clinical data are available through NIDDK Central Repository portal (https://repository.niddk.nih.gov/studies), and intermediate data/results are available in the Supporting Data Values file. All interested investigators in this study and related data are encouraged to contact authors for the integrated data and possible collaboration.

## Author contributions

LPZ, ÅL, and GKP researched and analyzed the data and wrote the manuscript. JSS was an investigator who led the DPT-1 trial and contributed to the interpretation of results. WWK reviewed the manuscript and expanded on implications of findings in the context of *DRB3* binding peptides. DEG led the team of RW, CWP, and WCN in the next-generation sequencing and researched data. AKM and GPB carried out graphical representations of select DR molecules and contributed to the Discussion section of the manuscript. LPZ and ÅL are guarantors of this work, had full access to all the data, and take full responsibility for the integrity of data and accuracy of data analysis.

## Supplementary Material

Supplemental data

Supporting data values

## Figures and Tables

**Table 1:**
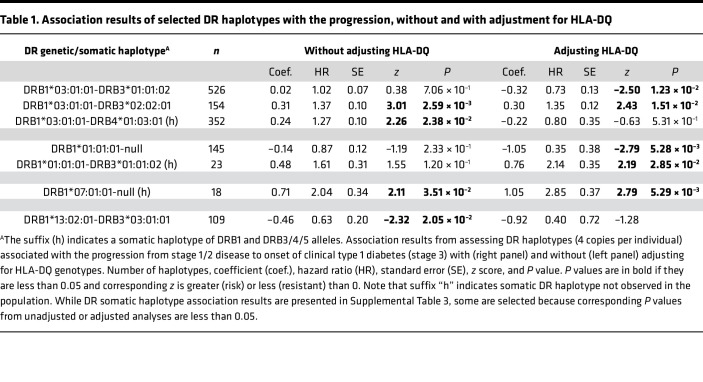
Association results of selected DR haplotypes with the progression, without and with adjustment for HLA-DQ

**Table 2:**
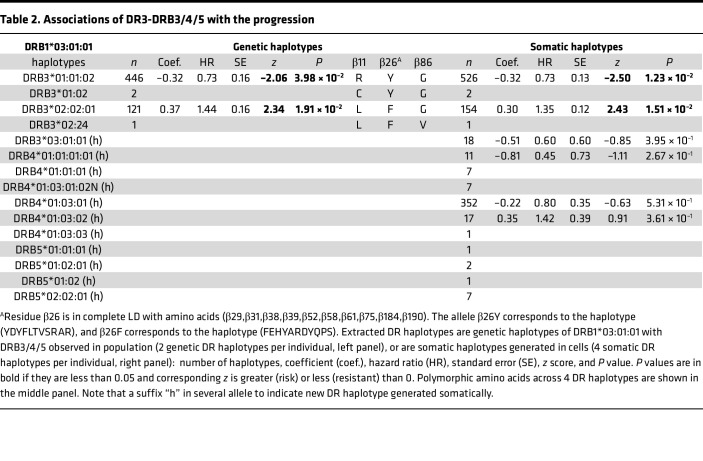
Associations of DR3-DRB3/4/5 with the progression

**Table 3:**
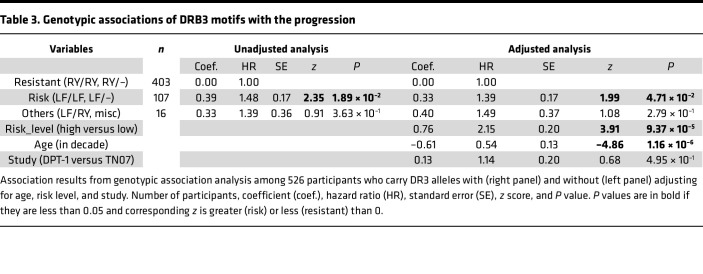
Genotypic associations of DRB3 motifs with the progression

## Version 1 03/04/2025

In-Press Preview

## Version 2 04/08/2025

Electronic publication
