# Supplementary material for: Two DRB3 residues predictively associate with the progression to type 1 diabetes among DR3 carriers
Source: JCI Insight. 2025 Mar 4;10(7):e184348. doi: 10.1172/jci.insight.184348 (PMC11981622; doi:10.1172/jci.insight.184348)
Supplement: Supplemental data [file jciinsight-10-184348-s222.pdf]

Supplementary Materials

Figure S1. Given two nearly monomorphic DRA alleles per subject, heterozygous *DRB1* and *DRB3/4/5* genes could produce as many as four distinct DRB molecules on the cell membrane of an Antigen-Presenting Cell: two DRB1 molecules and two DRB3/4/5 molecules. The protein chain from a *DRB1* gene is termed β1, while that from a DRB3/4/5 gene is termed β345.

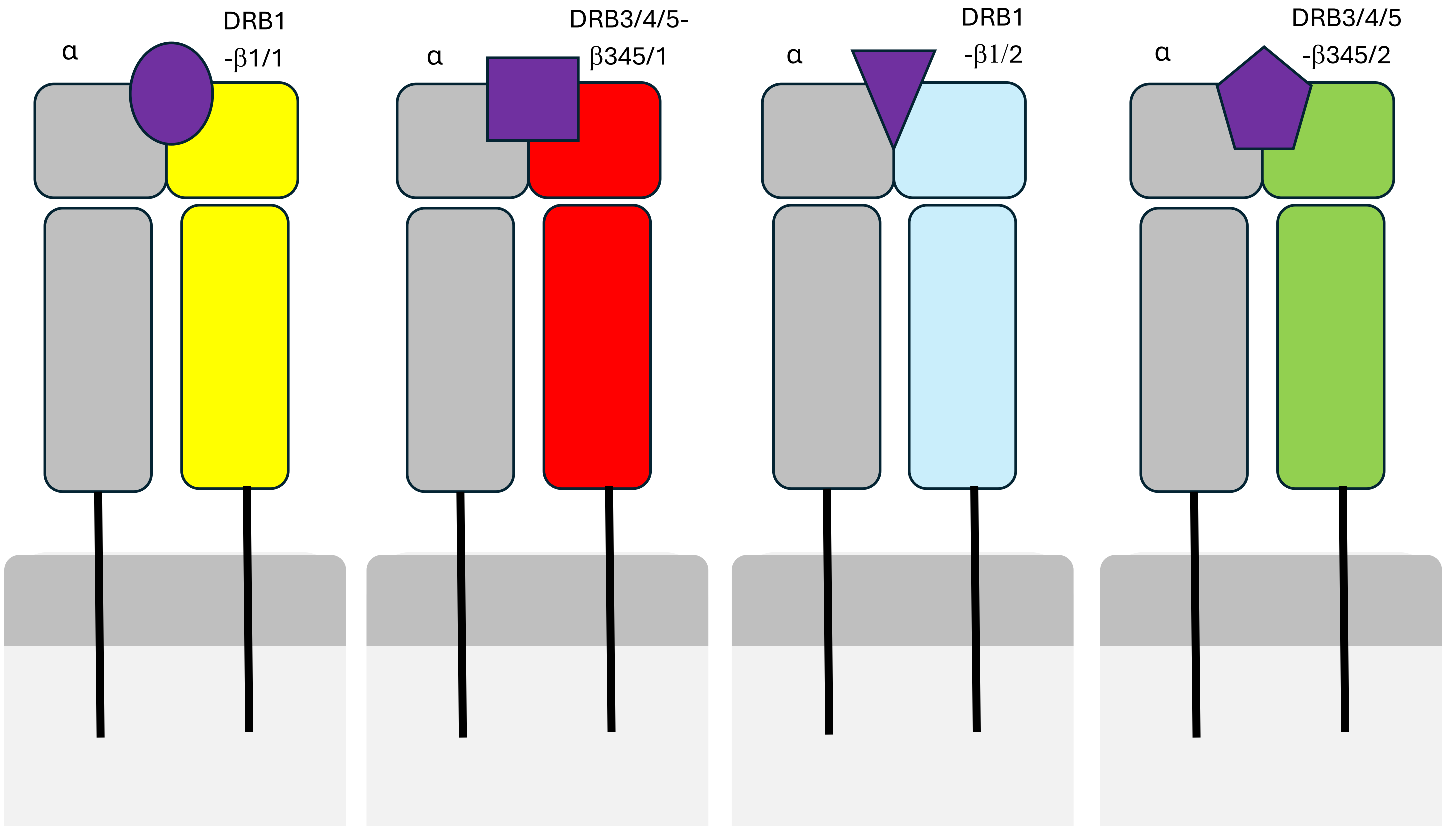

Figure S2. Amino acid sequences of HLA-DR3 and select DRB3 alleles, from the signal sequence to the intra-cytoplasmic tail; the former is in linkage disequilibrium with the latter. Notations, symbols and colorings are explained at the end of the sequences.

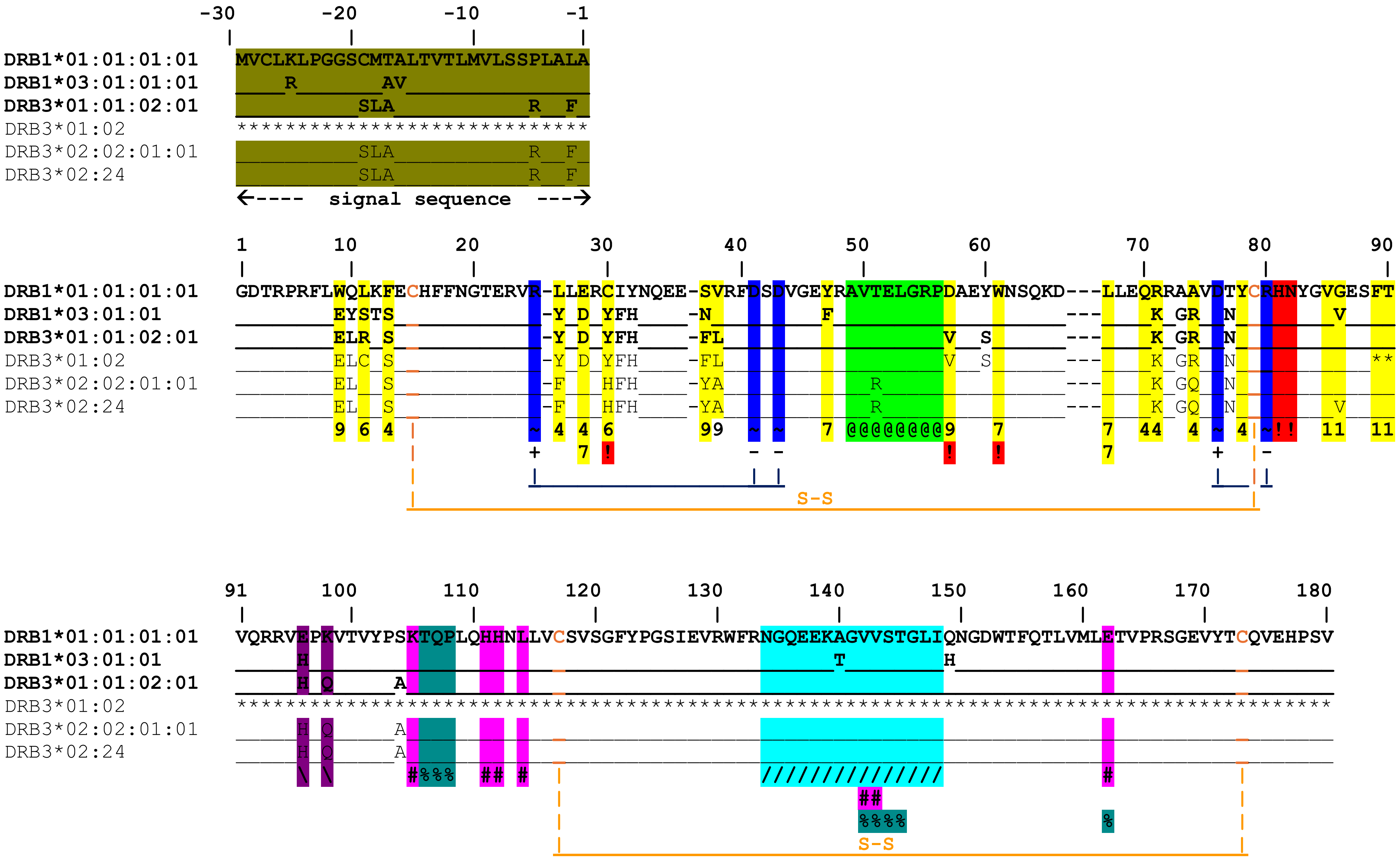
-30 -20 -10 -1
DRB1*01:01:01:01 MVCLKLPGGSCMTALTVTLMVLSSPLALA
DRB1*03:01:01:01 R AV
DRB3*01:01:02:01 SLA R F
DRB3*01:02 *****************************
DRB3*02:02:01:01 SLA R F
DRB3*02:24 SLA R F
signal sequence
1 10 20 30 40 50 60 70 80 90
DRB1*01:01:01:01 GDTRPRFLWQLKFECHFFNGTERVR-LLERCIYNQEE-SVRFDSDVGEYRAVTELGRPDAEYWNSQKD---LLEQRRAAVDTYCRHNYGVGESFT
DRB1*03:01:01 EYSTS -Y D YFH -N F --- K GR N V
DRB3*01:01:02:01 ELR S -Y D YFH -FL V S --- K GR N
DRB3*01:02 ELC S -Y D YFH -FL V S --- K GR N **
DRB3*02:02:01:01 EL S -F HFH -YA R --- K GQ N
DRB3*02:24 EL S -F HFH -YA R --- K GQ N V
9 6 4 ~ 4 4 6 99 ~ ~ 7 @@@@@@@@@9 7 7 44 4 ~ 4 ~!! 11 11
+ 7 ! - - ! ! 7 + -
S-S
91 100 110 120 130 140 150 160 170 180
DRB1*01:01:01:01 VQRRVEPKVTVYPSKTQPLQHHNLLVCSVSGFYPGSIEVRWFRNGQEEKAGVVSTGLIQNGDWTFQTLVMLETVPRSGEVYTCQVEHPSV
DRB1*03:01:01 H T H
DRB3*01:01:02:01 H Q A
DRB3*01:02 **
DRB3*02:02:01:01 H Q A
DRB3*02:24 H Q A
\ \ #%%% ## # /////////////// #
##
%%%% %
S-S

```
                  181     190       200       210       220       230     237
                  |       |         |         |         |         |       |
DRB1*01:01:01:01  TSPLTVEWRARSESAQSKMLSGVGGFVLGLLFLGAGLFIYFRNQKGHSGLQPTGFLS
DRB1*03:01:01     ____________________________________________________R____
DRB3*01:01:02:01  ___A_____________________________________________________
DRB3*01:02        *********************************************************
DRB3*02:02:01:01  ________S________________________________________________
DRB3*02:24        ________S________________________________________________
                     \  \ \                     cc  c      c  U
                        ~ ~
                        - +
```

Notes:
*: unknown residue
_: identical residue
-: deletion, no residue. The deletions between β25-β26 and β36-β37 are found in this latest release of HLA-DRB1 sequences, while the three deletions between β66-β67 are found in the latest release of HLA-DRB3 sequences (2024-04-10see details of release below). In order to be consistent with both conventions we have incorporated them both in the depiction of sequences here, even though none of the alleles concerned has an insertion in the specified positions.

Code: Identical to what appears in Bondinas et al, *Immunogenetics*, 2007 (reference 12, in the main text) with certain additions: brown-gray, signal peptide; yellow, participating in one of the five anchoring pockets, (noted below with respective numbers 1, 4, 6, 7 and 9); blue, intra-chain charge-charge attraction (noted below with **~** and respective charge sign); red, if appropriate polar residue, forms inter-chain hydrogen bond to carbonyl/amide group of bound antigenic peptide (noted below with a !). Only a fraction of such interactions are shown; we note particularly the many such interactions in the region β128-169 that stabilize the twisted β-sheet, β134-148, that participates in CD4 and LAG-3 binding; bright green, part of β49-56 homodimerization patch (noted below with **@**); pink, participating in cognate TCR-induced homodimerization (noted below with **#**); turquoise, participating in CD4 binding (noted below with **/**); dark cyan, participating in interactions with LAG-3 dimer (noted below with %, Reference 1, below); purple, residue interacting with HLA-DM in the endosome (pH = 5.5) for the release of bound CLIP, when complex is in the endosomal compartment (noted below with \); brick, ubiquitination site of HLA II by March-1 ubiquitin ligase (noted at the bottom with **U**); gray, intramembranous region; light gray, cell membrane and intra-cytoplasmic residues participating in stabilization in cholesterol rafts (noted below with **c)**. With the exception of the β49-56 homodimerization patch, all other properties of residues in the β-chain, have counterparts in the α-chain. The participation of residues from each chain depends on the interaction concerned: for peptide antigen binding the participation in the formation of the groove and the respective pockets is nearly even; however, for other interactions, such as with HLA-DM, the participation is mostly from residues of the α-chain (Pos et al., *Cell*, 2012, reference 14, in the main text). Interaction of HLA-DR with HLA-II invariant chain (Ii or CD74) is exclusively with the α-chain (Reference 2, below).

```
Source:
# file: DRB3_prot.txt
# date: 2024-04-10
```

version: IPD-IMGT/HLA 3.56.0
origin: https://github.com/ANHIG/IMGTHLA/blob/Latest/alignments/DRB3_prot.txt
repository: https://raw.githubusercontent.com/ANHIG/IMGTHLA/Latest/alignments/DRB3_prot.txt

Figure S3. Figure depicting most of the interchain HLA-DR1—HLA-DM β2–β2 domain interactions, as shown in the respective crystal structure at pH = 5.5. Only the pertinent portions of these two domains are shown. The types of interaction are detailed in the respective Table. Secondary structures shown as line ribbons (α-helices in red, β-sheets in turquoise, β-turns in green, and random coils in light gray). The interacting residues are in stick (outlined and mentioned in the original publication, below) or ball and stick form (delineated here) with the following atomic color code: carbon, gray; oxygen, red; nitrogen, cyan; hydrogen, white; sulfur, yellow (14).

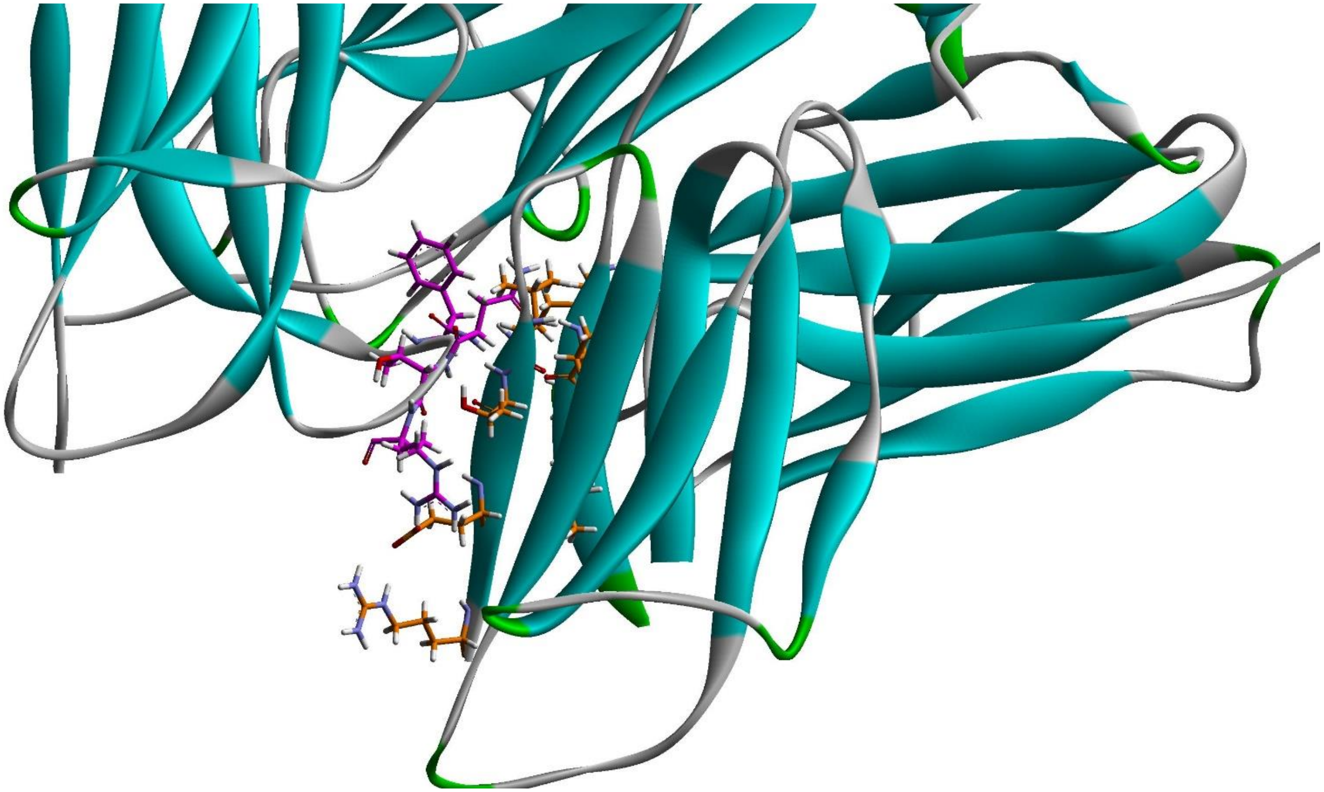

Figure S4. Fan-representation of 73 DR genetic haplotypes in 5 identified clusters.

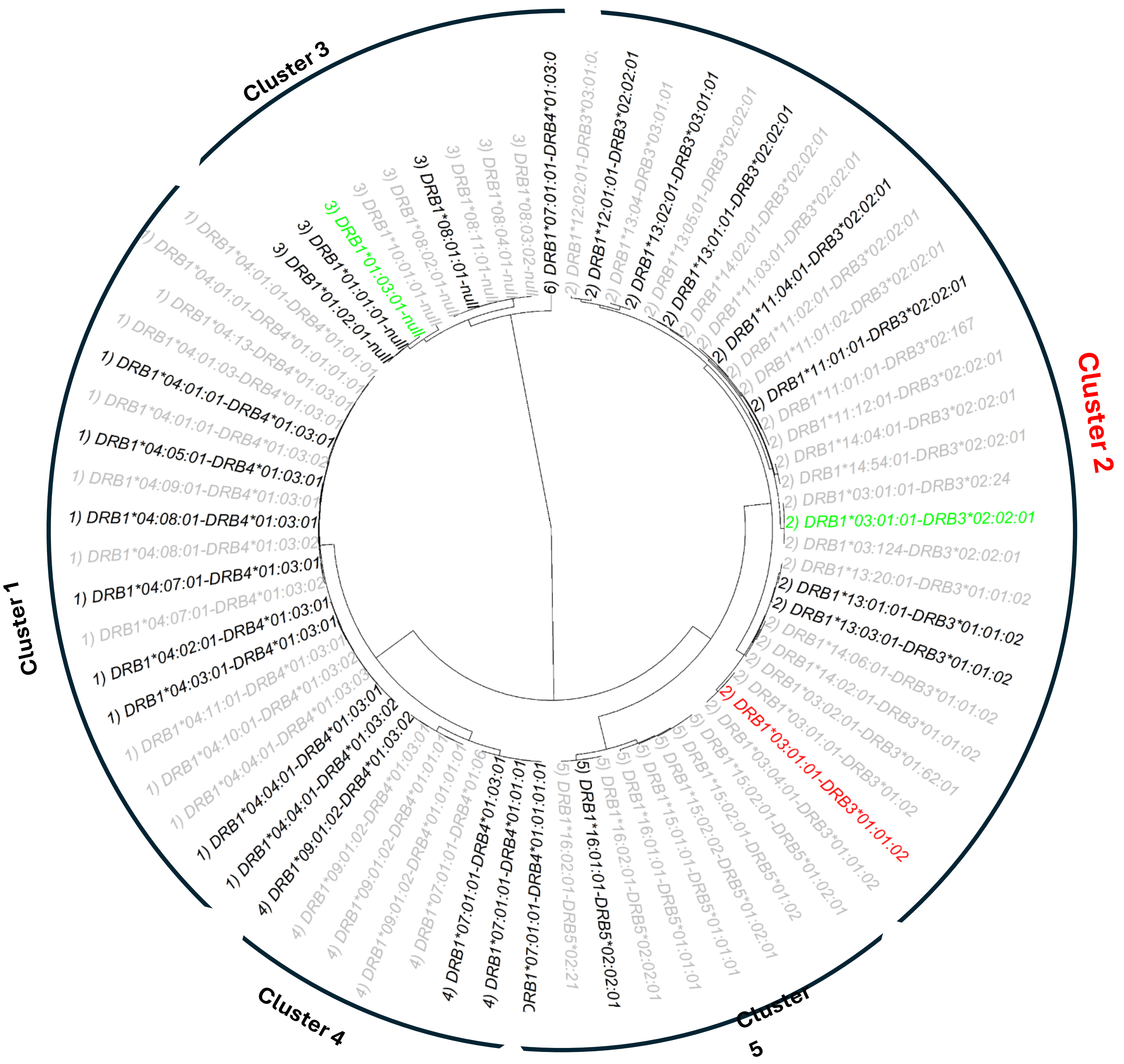

Figure S5. Fan-representation of all 249 DR somatic haplotypes (heterodimers) in 10 identified clusters.

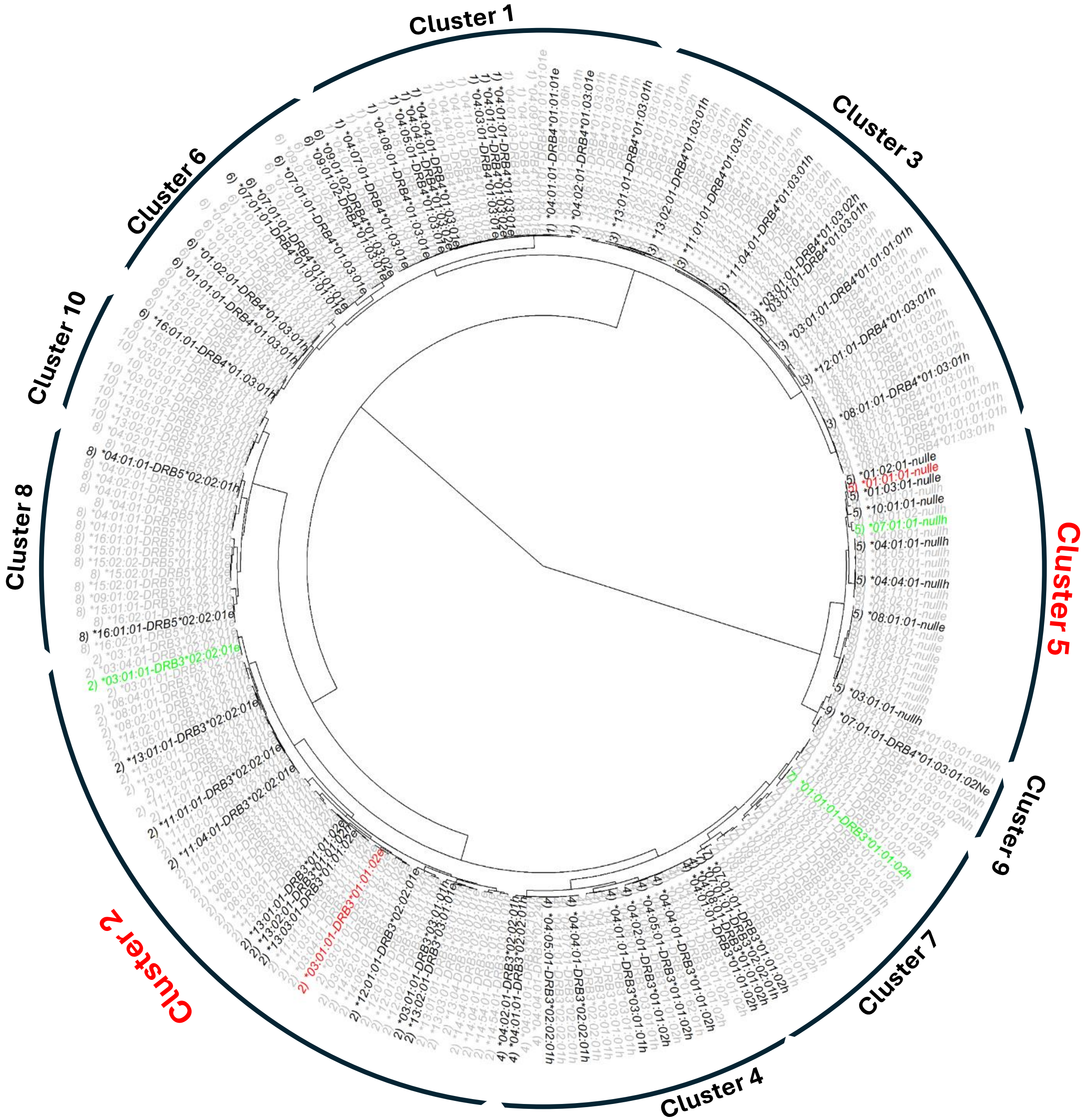

Table S1. Association results from allelic association analysis on HLA-DRB1 and HLA-DRB3/4/5 with the progression with (right panel) or without (left panel) adjustment for HLA-DQ genotypes: number of haplotypes, coefficient, Hazard ratio, standard error, Z-score and p-value. P-values are highlighted green or yellow for p-value less than 0.05 and corresponding HR is greater (risk) or less (resistant) than 1, respectively.

		Without Adjusting for HLA-DQ					Adjusting for HLA-DQ				
DRB1/3/4/5	n	coef	HR	SE	Z	p	coef	HR	SE	Z	p
DRB1*01:01:01	125	-0.13	0.88	0.15	-0.85	3.95E-01	-0.46	0.63	0.45	-1.02	3.07E-01
DRB1*01:02:01	20	-0.04	0.96	0.39	-0.11	9.15E-01	0.26	1.30	0.45	0.59	5.54E-01
DRB1*01:03:01	14	0.52	1.68	0.38	1.35	1.77E-01	0.90	2.46	0.41	2.22	2.67E-02
DRB1*03:01:01	570	0.18	1.20	0.09	2.12	3.39E-02	-0.02	0.98	0.40	-0.06	9.52E-01
DRB1*04:01:01	750	0.03	1.03	0.08	0.42	6.74E-01	0.01	1.01	0.11	0.09	9.28E-01
DRB1*04:02:01	54	0.30	1.35	0.19	1.61	1.06E-01	0.18	1.20	0.20	0.91	3.64E-01
DRB1*04:03:01	12	0.38	1.46	0.45	0.83	4.05E-01	0.17	1.19	0.46	0.38	7.06E-01
DRB1*04:04:01	167	0.07	1.08	0.14	0.53	5.97E-01	-0.12	0.88	0.15	-0.84	4.04E-01
DRB1*04:05:01	70	0.17	1.19	0.21	0.84	4.01E-01	0.07	1.08	0.26	0.28	7.79E-01
DRB1*04:07:01	28	-0.37	0.69	0.45	-0.82	4.15E-01	-0.34	0.71	0.45	-0.76	4.49E-01
DRB1*04:08:01	26	0.02	1.03	0.32	0.08	9.38E-01	0.26	1.30	0.35	0.76	4.47E-01
DRB1*07:01:01	129	-0.15	0.86	0.17	-0.90	3.69E-01	-0.04	0.96	0.50	-0.08	9.38E-01
DRB1*08:01:01	46	0.07	1.07	0.24	0.27	7.86E-01	0.27	1.30	0.26	1.02	3.06E-01
DRB1*09:01:02	32	-0.03	0.97	0.30	-0.10	9.19E-01	0.13	1.14	0.32	0.40	6.87E-01
DRB1*11:01:01	42	-0.55	0.58	0.31	-1.78	7.50E-02	-0.61	0.54	0.37	-1.64	1.02E-01
DRB1*11:04:01	16	-0.43	0.65	0.58	-0.74	4.61E-01	-0.35	0.71	0.62	-0.56	5.77E-01
DRB1*12:01:01	19	0.46	1.59	0.36	1.30	1.94E-01	0.77	2.17	0.40	1.95	5.09E-02
DRB1*13:01:01	76	-0.18	0.83	0.23	-0.80	4.21E-01	-0.58	0.56	0.95	-0.61	5.40E-01
DRB1*13:02:01	103	-0.47	0.63	0.20	-2.27	2.30E-02	-0.29	0.75	0.44	-0.64	5.20E-01
DRB1*13:03:01	16	-0.11	0.90	0.50	-0.22	8.27E-01	0.16	1.17	0.53	0.30	7.64E-01
DRB1*16:01:01	26	-0.41	0.67	0.38	-1.06	2.89E-01	-0.20	0.82	0.40	-0.49	6.25E-01
DRB1 (<10 obs)	87	-0.34	0.71	0.25	-1.39	1.66E-01	-0.22	0.80	0.26	-0.84	4.00E-01

DRB3*01:01:02	510	0.04	1.04	0.09	0.40	6.86E-01	-0.23	0.79	0.14	-1.64	1.00E-01
DRB3*02:02:01	262	0.12	1.12	0.11	1.10	2.73E-01	0.23	1.26	0.13	1.71	8.65E-02
DRB3*03:01:01	104	-0.47	0.63	0.20	-2.28	2.24E-02	-0.30	0.74	0.44	-0.66	5.07E-01
DRB4*01:01:01	59	0.08	1.09	0.22	0.39	7.00E-01	0.19	1.21	0.25	0.76	4.45E-01
DRB4*01:01:01:01	27	-0.35	0.70	0.38	-0.93	3.54E-01	-0.21	0.81	0.42	-0.50	6.15E-01
DRB4*01:03:01	1118	0.07	1.08	0.08	0.90	3.66E-01	-0.11	0.90	0.16	-0.66	5.09E-01
DRB4*01:03:01:02N	12	-0.50	0.61	0.71	-0.71	4.79E-01	-0.36	0.70	0.72	-0.50	6.15E-01
DRB4*01:03:02	57	0.08	1.08	0.22	0.35	7.27E-01	0.13	1.14	0.23	0.56	5.76E-01
DRB5*02:02:01	26	-0.38	0.68	0.38	-0.99	3.21E-01	-0.16	0.85	0.40	-0.40	6.91E-01
null	230	-0.09	0.91	0.12	-0.77	4.38E-01	0.14	1.15	0.21	0.66	5.12E-01
DRB345 (<10 obs)	23	-0.10	0.91	0.45	-0.21	8.32E-01	-0.04	0.96	0.46	-0.08	9.33E-01

Table S2. Association results from haplotypic association analysis on all possible HLA-DR haplotypes with the progression: number of haplotypes, coefficient, hazard ratio, standard error, Z-score and p-value. P-values are highlighted green or yellow for p-value less than 0.05 and corresponding HR is greater (risk) or less (resistant) than 1, respectively. Note that for many uncommon DR haplotypes with fewer than 10 observations, association statistics were not computable, since there were no T1D event(s) observed (coef<0), or there is only one T1D event associated with a single DR haplotype (coef>0). This result was obtained earlier by us [5].

seq	DRB1-DRB3/4/5		Adjusted Age & Risk Level					Adjusting DQ, Age & Risk Level				
		n	coef	HR	SE	Z	p	coef	HR	SE	Z	p
1	DRB1*01:01-Null	125	-0.13	0.88	0.15	-0.86	3.92E-01	-0.39	0.68	0.32	-1.23	2.17E-01
2	DRB1*01:02-Null	20	-0.04	0.96	0.39	-0.10	9.17E-01	-0.03	0.97	0.42	-0.06	9.51E-01
3	DRB1*01:03-Null	14	0.52	1.68	0.38	1.35	1.76E-01	0.84	2.32	0.41	2.06	3.94E-02
4	DRB1*03:01-DRB3*01:01	449	0.04	1.05	0.09	0.48	6.32E-01	-0.32	0.73	0.16	-2.06	3.98E-02
5	DRB1*03:01-DRB3*02:02	121	0.46	1.59	0.14	3.28	1.05E-03	0.37	1.44	0.16	2.34	1.91E-02
6	DRB1*04:01-DRB4*01:03	747	0.04	1.04	0.08	0.46	6.42E-01	0.02	1.02	0.11	0.21	8.37E-01
7	DRB1*04:02-DRB4*01:03	54	0.30	1.35	0.19	1.61	1.07E-01	0.16	1.17	0.19	0.82	4.11E-01
8	DRB1*04:03-DRB4*01:03	12	0.38	1.46	0.45	0.83	4.05E-01	0.20	1.22	0.45	0.44	6.58E-01
9	DRB1*04:04-DRB4*01:03	167	0.07	1.08	0.14	0.52	6.00E-01	-0.09	0.91	0.15	-0.62	5.32E-01
10	DRB1*04:05-DRB4*01:03	70	0.17	1.19	0.21	0.84	4.02E-01	0.15	1.16	0.27	0.55	5.84E-01
11	DRB1*04:07-DRB4*01:03	28	-0.37	0.69	0.45	-0.82	4.14E-01	-0.35	0.70	0.45	-0.78	4.37E-01
12	DRB1*04:08-DRB4*01:03	26	0.02	1.02	0.32	0.08	9.40E-01	-0.16	0.85	0.73	-0.21	8.31E-01
13	DRB1*07:01-DRB4*01:01	73	-0.02	0.98	0.21	-0.08	9.34E-01	0.33	1.39	0.35	0.96	3.40E-01
14	DRB1*07:01-DRB4*01:03	55	-0.41	0.67	0.28	-1.43	1.52E-01	-0.43	0.65	0.37	-1.17	2.43E-01
15	DRB1*08:01-Null	46	0.06	1.07	0.24	0.27	7.87E-01	0.30	1.35	0.39	0.75	4.50E-01
16	DRB1*09:01-DRB4*01:03	24	0.04	1.04	0.34	0.11	9.12E-01	0.13	1.14	0.37	0.35	7.23E-01
17	DRB1*11:01-DRB3*02:02	44	-0.50	0.61	0.29	-1.70	8.83E-02	-0.33	0.72	0.36	-0.91	3.63E-01
18	DRB1*11:04-DRB3*02:02	16	-0.43	0.65	0.58	-0.74	4.61E-01	-0.21	0.81	0.62	-0.34	7.32E-01
19	DRB1*12:01-DRB3*02:02	19	0.46	1.59	0.36	1.30	1.94E-01	0.96	2.60	0.40	2.37	1.77E-02
20	DRB1*13:01-DRB3*01:01	44	-0.03	0.97	0.30	-0.09	9.26E-01	0.17	1.18	0.43	0.40	6.92E-01
21	DRB1*13:01-DRB3*02:02	32	-0.34	0.71	0.34	-1.00	3.16E-01	-0.30	0.74	0.45	-0.68	4.98E-01
22	DRB1*13:02-DRB3*03:01	103	-0.47	0.63	0.20	-2.27	2.31E-02	<<0				

23	DRB1*13:03-DRB3*01:01	16	-0.11	0.90	0.50	-0.22	8.26E-01	0.18	1.20	0.54	0.34	7.33E-01
24	DRB1*16:01-DRB5*02:02	25	-0.37	0.69	0.38	-0.96	3.35E-01	-0.92	0.40	0.80	-1.15	2.51E-01
	Uncommon haplotypes*	102	-0.34	0.71	0.22	-1.53	1.26E-01	-0.31	0.73	0.28	-1.13	2.56E-01

* Including all haplotypes with fewer than 9 observations in the iCohort

Table S3. Association results from haplotypic association analysis on all possible HLA-DR haplotypes with the progression: number of haplotypes, coefficient, Hazard ratio, standard error, Z-score and p-value. P-values are highlighted green or yellow for p-value less than 0.05 and corresponding HR is greater (risk) or less (resistant) than 1, respectively. Note that for many uncommon DR haplotypes with fewer than 10 observations, association statistics were not computable, since there were no T1D event(s) observed (coef<0), or there is only one T1D event associated with a single DR haplotype (coef>0). Note that a suffix "h" in several allele to indicate new DR haplotype generated somatically.

seq	DR haplotype	n	coef	HR	SE	Z	p
1	DRB1*01:01:01-DRB3*01:01:02h	23	0.64	1.90	0.31	2.09	3.63E-02
2	DRB1*01:01:01-DRB3*01:02h	1	<0				
3	DRB1*01:01:01-DRB3*02:02:01h	6	-0.09	0.91	0.58	-0.15	8.78E-01
4	DRB1*01:01:01-DRB3*03:01:01h	5	<0				
5	DRB1*01:01:01-DRB4*01:01:01h	3	0.97	2.64	0.71	1.37	1.71E-01
6	DRB1*01:01:01-DRB4*01:03:01h	64	-0.09	0.91	0.22	-0.41	6.82E-01
7	DRB1*01:01:01-DRB4*01:03:02h	2	<0				
8	DRB1*01:01:01-DRB5*01:01:01h	1	<0				
9	DRB1*01:01:01-null	145	-0.08	0.93	0.12	-0.64	5.22E-01
10	DRB1*01:02:01-DRB3*01:01:02h	1	<0				
11	DRB1*01:02:01-DRB3*02:02:01h	1	<0				
12	DRB1*01:02:01-DRB4*01:03:01h	11	0.36	1.44	0.50	0.72	4.71E-01
13	DRB1*01:02:01-null	27	0.15	1.16	0.25	0.58	5.65E-01
14	DRB1*01:03:01-DRB3*01:01:02h	1	1.04	2.84	1.00	1.04	2.98E-01
15	DRB1*01:03:01-DRB4*01:01:01:01h	1	>0				
16	DRB1*01:03:01-DRB4*01:03:01h	8	0.82	2.27	0.50	1.63	1.03E-01
17	DRB1*01:03:01-DRB4*01:03:02h	1	<0				
18	DRB1*01:03:01-null	17	0.50	1.65	0.29	1.73	8.38E-02
19	DRB1*03:01:01-DRB3*01:01:02	526	-0.08	0.92	0.07	-1.15	2.52E-01
28	DRB1*03:01:01-DRB3*01:02	2	<0				
21	DRB1*03:01:01-DRB3*02:02:01	154	0.23	1.26	0.11	2.22	2.64E-02
30	DRB1*03:01:01-DRB3*02:24	1	<0				

22	DRB1*03:01:01-DRB3*03:01:01h	18	-0.47	0.62	0.58	-0.82	4.14E-01
24	DRB1*03:01:01-DRB4*01:01:01:01h	11	-0.75	0.47	0.71	-1.06	2.88E-01
25	DRB1*03:01:01-DRB4*01:01:01h	7	0.91	2.48	0.50	1.80	7.15E-02
26	DRB1*03:01:01-DRB4*01:03:01:02Nh	7	<0				
20	DRB1*03:01:01-DRB4*01:03:01h	352	-0.02	0.98	0.11	-0.18	8.55E-01
23	DRB1*03:01:01-DRB4*01:03:02h	17	0.35	1.41	0.38	0.90	3.66E-01
31	DRB1*03:01:01-DRB4*01:03:03h	1	0.48	1.62	1.00	0.48	6.31E-01
32	DRB1*03:01:01-DRB5*01:01:01h	1	<0				
29	DRB1*03:01:01-DRB5*01:02:01h	2	<0				
33	DRB1*03:01:01-DRB5*01:02h	1	<0				
27	DRB1*03:01:01-DRB5*02:02:01h	7	0.15	1.17	0.58	0.27	7.91E-01
34	DRB1*03:01:01-nullh	33	0.37	1.45	0.27	1.36	1.73E-01
35	DRB1*03:02:01-DRB3*01:62:01	1	<0				
36	DRB1*03:02:01-DRB4*01:03:01h	1	<0				
37	DRB1*03:04:01-DRB3*01:01:02	1	<0				
38	DRB1*03:04:01-DRB3*02:02:01h	1	<0				
39	DRB1*03:124-DRB3*02:02:01	1	<0				
40	DRB1*03:124-DRB4*01:03:01h	1	<0				
41	DRB1*04:01:01-DRB3*01:01:02h	201	-0.14	0.87	0.13	-1.05	2.95E-01
42	DRB1*04:01:01-DRB3*01:02h	1	<0				
43	DRB1*04:01:01-DRB3*02:02:01h	100	0.06	1.06	0.17	0.36	7.20E-01
44	DRB1*04:01:01-DRB3*02:167h	1	>0				
45	DRB1*04:01:01-DRB3*03:01:01h	41	-0.16	0.85	0.32	-0.49	6.23E-01
47	DRB1*04:01:01-DRB4*01:01:01	31	0.00	1.00	0.27	0.01	9.94E-01
46	DRB1*04:01:01-DRB4*01:01:01:01	1	0.79	2.20	1.00	0.79	4.31E-01
49	DRB1*04:01:01-DRB4*01:03:01	997	0.01	1.01	0.05	0.22	8.29E-01
48	DRB1*04:01:01-DRB4*01:03:01:02Nh	2	0.83	2.30	1.00	0.83	4.07E-01
50	DRB1*04:01:01-DRB4*01:03:02	18	0.39	1.47	0.32	1.21	2.26E-01
51	DRB1*04:01:01-DRB4*01:06h	1	>0				

52	DRB1*04:01:01-DRB5*01:01:01h	2	<0				
53	DRB1*04:01:01-DRB5*01:02:01h	2	<0				
54	DRB1*04:01:01-DRB5*01:02h	3	<0				
55	DRB1*04:01:01-DRB5*02:02:01h	11	-0.18	0.83	0.58	-0.31	7.54E-01
56	DRB1*04:01:01-nullh	88	0.06	1.06	0.19	0.31	7.53E-01
57	DRB1*04:01:03-DRB4*01:03:01	2	<0				
58	DRB1*04:02:01-DRB3*01:01:02h	10	0.62	1.85	0.45	1.37	1.71E-01
59	DRB1*04:02:01-DRB3*02:02:01h	11	0.24	1.27	0.38	0.62	5.35E-01
60	DRB1*04:02:01-DRB3*03:01:01h	2	0.60	1.83	1.00	0.60	5.48E-01
61	DRB1*04:02:01-DRB4*01:01:01h	1	<0				
62	DRB1*04:02:01-DRB4*01:03:01	75	0.05	1.05	0.12	0.37	7.09E-01
63	DRB1*04:02:01-DRB5*01:01:01h	1	>0				
64	DRB1*04:02:01-DRB5*02:02:01h	1	<0				
65	DRB1*04:02:01-nullh	7	0.35	1.42	0.58	0.61	5.44E-01
66	DRB1*04:03:01-DRB3*01:01:02h	5	0.55	1.74	0.58	0.95	3.42E-01
67	DRB1*04:03:01-DRB3*02:02:01h	3	0.21	1.24	1.00	0.21	8.33E-01
68	DRB1*04:03:01-DRB4*01:03:01	16	0.01	1.01	0.33	0.03	9.80E-01
69	DRB1*04:04:01-DRB3*01:01:02h	61	-0.09	0.91	0.21	-0.45	6.49E-01
70	DRB1*04:04:01-DRB3*02:02:01h	33	0.16	1.17	0.28	0.56	5.76E-01
71	DRB1*04:04:01-DRB3*02:24h	1	<0				
72	DRB1*04:04:01-DRB3*03:01:01h	7	0.45	1.57	1.00	0.45	6.55E-01
73	DRB1*04:04:01-DRB4*01:01:01h	1	<0				
75	DRB1*04:04:01-DRB4*01:03:01	192	-0.11	0.90	0.11	-0.93	3.51E-01
74	DRB1*04:04:01-DRB4*01:03:01:02Nh	1	<0				
76	DRB1*04:04:01-DRB4*01:03:02	25	0.07	1.08	0.32	0.23	8.16E-01
77	DRB1*04:04:01-DRB4*01:03:03	1	0.48	1.62	1.00	0.48	6.31E-01
78	DRB1*04:04:01-DRB5*02:21h	1	0.64	1.90	1.00	0.64	5.20E-01
79	DRB1*04:04:01-nullh	11	-0.70	0.50	0.58	-1.21	2.27E-01
80	DRB1*04:05:01-DRB3*01:01:02h	21	0.16	1.18	0.38	0.42	6.72E-01
81	DRB1*04:05:01-DRB3*01:62:01h	1	<0				

82	DRB1*04:05:01-DRB3*02:02:01h	15	-0.13	0.88	0.50	-0.26	7.98E-01
83	DRB1*04:05:01-DRB3*03:01:01h	4	0.75	2.11	0.71	1.05	2.92E-01
84	DRB1*04:05:01-DRB4*01:01:01h	3	<0				
85	DRB1*04:05:01-DRB4*01:03:01	88	0.03	1.03	0.14	0.23	8.20E-01
86	DRB1*04:05:01-nullh	8	0.16	1.17	0.58	0.27	7.85E-01
87	DRB1*04:07:01-DRB3*01:01:02h	5	0.87	2.40	1.01	0.87	3.84E-01
88	DRB1*04:07:01-DRB3*02:02:01h	8	0.66	1.94	0.58	1.15	2.52E-01
89	DRB1*04:07:01-DRB4*01:01:01h	2	<0				
90	DRB1*04:07:01-DRB4*01:03:01	31	-0.51	0.60	0.41	-1.23	2.19E-01
91	DRB1*04:07:01-DRB4*01:03:02	8	-0.16	0.85	0.71	-0.23	8.20E-01
92	DRB1*04:07:01-DRB5*02:02:01h	1	<0				
93	DRB1*04:07:01-nullh	1	<0				
94	DRB1*04:08:01-DRB3*01:01:02h	10	0.39	1.48	0.45	0.87	3.87E-01
95	DRB1*04:08:01-DRB3*03:01:01h	2	0.28	1.33	1.01	0.28	7.80E-01
96	DRB1*04:08:01-DRB4*01:03:01	36	0.12	1.13	0.23	0.53	5.94E-01
97	DRB1*04:08:01-DRB4*01:03:02	1	>0				
98	DRB1*04:08:01-nullh	3	<0				
99	DRB1*04:09:01-DRB3*01:01:02h	1	<0				
100	DRB1*04:09:01-DRB4*01:03:01	1	<0				
101	DRB1*04:10:01-DRB4*01:03:02	1	<0				
102	DRB1*04:10:01-nullh	1	<0				
103	DRB1*04:11:01-DRB3*02:02:01h	1	<0				
104	DRB1*04:11:01-DRB4*01:03:01	3	<0				
105	DRB1*04:11:01-nullh	2	<0				
106	DRB1*04:13-DRB4*01:03:01	2	<0				
107	DRB1*07:01:01-DRB3*01:01:02h	28	0.04	1.04	0.36	0.11	9.16E-01
108	DRB1*07:01:01-DRB3*02:02:01h	12	0.10	1.10	0.58	0.17	8.67E-01
109	DRB1*07:01:01-DRB3*03:01:01h	9	-0.08	0.92	0.71	-0.12	9.05E-01
111	DRB1*07:01:01-DRB4*01:01:01	52	0.28	1.32	0.24	1.17	2.41E-01
110	DRB1*07:01:01-DRB4*01:01:01:01	25	-0.26	0.77	0.41	-0.64	5.25E-01
113	DRB1*07:01:01-DRB4*01:03:01	98	-0.17	0.85	0.16	-1.03	3.05E-01

112	DRB1*07:01:01-DRB4*01:03:01:02N	12	-0.35	0.70	0.71	-0.49	6.22E-01
114	DRB1*07:01:01-DRB4*01:03:02h	3	<0				
115	DRB1*07:01:01-DRB4*01:06	1	>0				
116	DRB1*07:01:01-nullh	18	0.92	2.50	0.34	2.71	6.83E-03
117	DRB1*08:01:01-DRB3*01:01:02h	2	<0				
118	DRB1*08:01:01-DRB3*02:02:01h	1	<0				
119	DRB1*08:01:01-DRB3*03:01:01h	1	<0				
120	DRB1*08:01:01-DRB4*01:01:01:01h	3	1.05	2.85	0.71	1.47	1.41E-01
121	DRB1*08:01:01-DRB4*01:01:01h	3	1.16	3.20	0.71	1.63	1.02E-01
122	DRB1*08:01:01-DRB4*01:03:01h	28	0.21	1.24	0.31	0.70	4.83E-01
123	DRB1*08:01:01-DRB4*01:03:02h	1	<0				
124	DRB1*08:01:01-null	53	0.23	1.25	0.20	1.14	2.53E-01
125	DRB1*08:02:01-DRB3*02:02:01h	1	<0				
126	DRB1*08:02:01-DRB4*01:01:01:01h	2	<0				
127	DRB1*08:02:01-DRB4*01:03:01h	3	<0				
128	DRB1*08:02:01-DRB4*01:03:02h	1	<0				
129	DRB1*08:02:01-null	9	<0				
130	DRB1*08:03:02-DRB3*01:01:02h	1	<0				
131	DRB1*08:03:02-DRB4*01:03:01h	2	<0				
132	DRB1*08:03:02-null	3	<0				
133	DRB1*08:04:01-DRB3*02:02:01h	1	>0				
134	DRB1*08:04:01-DRB4*01:01:01:01h	1	<0				
135	DRB1*08:04:01-DRB4*01:03:01h	3	<0				
136	DRB1*08:04:01-null	5	0.36	1.44	1.00	0.36	7.18E-01
137	DRB1*08:11:01-DRB4*01:03:01h	1	<0				
138	DRB1*08:11:01-null	1	<0				
139	DRB1*09:01:02-DRB3*01:01:02h	7	0.41	1.51	0.58	0.70	4.81E-01
140	DRB1*09:01:02-DRB3*02:02:01h	6	<0				
141	DRB1*09:01:02-DRB3*03:01:01h	2	>0				
142	DRB1*09:01:02-DRB3*03:01:03h	1	<0				

144	DRB1*09:01:02-DRB4*01:01:01	8	-0.08	0.92	0.48	-0.17	8.63E-01
143	DRB1*09:01:02-DRB4*01:01:01:01	3	<0				
145	DRB1*09:01:02-DRB4*01:03:01	11	-0.04	0.96	0.50	-0.09	9.32E-01
146	DRB1*09:01:02-DRB4*01:03:02	24	0.28	1.32	0.29	0.94	3.48E-01
147	DRB1*09:01:02-DRB5*02:02:01h	1	0.84	2.31	1.00	0.84	4.02E-01
148	DRB1*09:01:02-nullh	1	<0				
149	DRB1*10:01:01-DRB3*01:01:02h	3	<0				
150	DRB1*10:01:01-DRB3*03:01:01h	1	<0				
151	DRB1*10:01:01-DRB4*01:01:01h	1	<0				
152	DRB1*10:01:01-DRB4*01:03:01h	1	<0				
153	DRB1*10:01:01-null	10	<0				
154	DRB1*11:01:01-DRB3*01:01:02h	6	<0				
155	DRB1*11:01:01-DRB3*02:02:01	47	-0.31	0.73	0.28	-1.11	2.65E-01
156	DRB1*11:01:01-DRB3*02:167	1	>0				
157	DRB1*11:01:01-DRB4*01:01:01:01h	1	<0				
158	DRB1*11:01:01-DRB4*01:03:01h	27	-0.31	0.73	0.36	-0.87	3.87E-01
159	DRB1*11:01:01-DRB4*01:03:02h	2	<0				
160	DRB1*11:01:02-DRB3*02:02:01	3	0.97	2.65	0.71	1.37	1.71E-01
161	DRB1*11:01:02-DRB4*01:03:01h	2	>0				
162	DRB1*11:01:02-DRB5*02:02:01h	1	<0				
163	DRB1*11:02:01-DRB3*02:02:01	5	>0				
164	DRB1*11:02:01-DRB4*01:03:01h	3	>0				
165	DRB1*11:02:01-DRB4*01:03:02h	1	<0				
166	DRB1*11:02:01-nullh	1	<0				
167	DRB1*11:03:01-DRB3*01:01:02h	2	<0				
168	DRB1*11:03:01-DRB3*02:02:01	7	-0.56	0.57	0.93	-0.60	5.47E-01
169	DRB1*11:03:01-DRB4*01:03:01h	3	0.07	1.08	1.00	0.07	9.42E-01
170	DRB1*11:04:01-DRB3*02:02:01	19	-0.36	0.70	0.53	-0.68	4.97E-01
171	DRB1*11:04:01-DRB4*01:01:01h	1	<0				
172	DRB1*11:04:01-DRB4*01:03:01h	10	0.00	1.00	0.58	0.00	9.99E-01
173	DRB1*11:04:01-DRB4*01:03:02h	2	<0				

174	DRB1*11:12:01-DRB3*02:02:01	1	>0				
175	DRB1*11:12:01-DRB4*01:03:01h	1	>0				
176	DRB1*12:01:01-DRB3*01:01:02h	2	0.77	2.17	1.00	0.77	4.41E-01
177	DRB1*12:01:01-DRB3*02:02:01	19	0.67	1.95	0.36	1.87	6.15E-02
178	DRB1*12:01:01-DRB3*03:01:01h	2	>0				
179	DRB1*12:01:01-DRB4*01:01:01:01h	1	<0				
180	DRB1*12:01:01-DRB4*01:03:01h	14	0.42	1.52	0.45	0.92	3.57E-01
181	DRB1*12:02:01-DRB3*03:01:03	1	<0				
182	DRB1*12:02:01-DRB4*01:03:01h	1	<0				
183	DRB1*13:01:01-DRB3*01:01:02	54	-0.05	0.95	0.27	-0.18	8.53E-01
184	DRB1*13:01:01-DRB3*02:02:01	36	-0.09	0.92	0.28	-0.31	7.58E-01
185	DRB1*13:01:01-DRB3*03:01:01h	1	<0				
186	DRB1*13:01:01-DRB4*01:01:01h	2	<0				
187	DRB1*13:01:01-DRB4*01:03:01:02Nh	1	>0				
188	DRB1*13:01:01-DRB4*01:03:01h	51	-0.02	0.98	0.27	-0.09	9.31E-01
189	DRB1*13:01:01-DRB5*02:02:01h	1	<0				
190	DRB1*13:01:01-nullh	6	0.15	1.16	0.71	0.21	8.34E-01
191	DRB1*13:02:01-DRB3*01:01:02h	15	-0.84	0.43	0.71	-1.19	2.35E-01
192	DRB1*13:02:01-DRB3*02:02:01h	7	1.58	4.85	0.51	3.12	1.80E-03
193	DRB1*13:02:01-DRB3*03:01:01	109	-0.10	0.91	0.19	-0.50	6.19E-01
194	DRB1*13:02:01-DRB4*01:01:01:01h	4	-0.49	0.61	1.00	-0.49	6.22E-01
195	DRB1*13:02:01-DRB4*01:01:01h	3	<0				
196	DRB1*13:02:01-DRB4*01:03:01h	56	0.08	1.09	0.26	0.32	7.49E-01
197	DRB1*13:02:01-DRB4*01:03:02h	4	>0				
198	DRB1*13:02:01-DRB5*02:02:01h	1	<0				
199	DRB1*13:02:01-nullh	7	<0				
200	DRB1*13:03:01-DRB3*01:01:02	19	-0.07	0.93	0.42	-0.16	8.70E-01
201	DRB1*13:03:01-DRB3*02:02:01h	1	>0				
202	DRB1*13:03:01-DRB3*03:01:01h	1	<0				

203	DRB1*13:03:01-DRB4*01:03:01:02Nh	1	<0				
204	DRB1*13:03:01-DRB4*01:03:01h	9	0.62	1.86	0.58	1.07	2.86E-01
205	DRB1*13:03:01-nullh	1	<0				
206	DRB1*13:04-DRB3*02:02:01h	1	<0				
207	DRB1*13:04-DRB3*03:01:01	1	<0				
208	DRB1*13:05:01-DRB3*01:01:02h	1	>0				
209	DRB1*13:05:01-DRB3*02:02:01	6	-0.68	0.51	0.88	-0.77	4.41E-01
210	DRB1*13:05:01-DRB4*01:03:01h	1	<0				
211	DRB1*13:05:01-DRB4*01:03:02h	1	<0				
212	DRB1*13:05:01-DRB5*02:02:01h	1	<0				
213	DRB1*13:20:01-DRB3*01:01:02	1	<0				
214	DRB1*13:20:01-DRB4*01:03:01h	1	<0				
215	DRB1*14:02:01-DRB3*01:01:02	1	<0				
216	DRB1*14:02:01-DRB3*02:02:01	2	>0				
217	DRB1*14:02:01-DRB4*01:03:01h	3	0.87	2.38	1.00	0.86	3.88E-01
218	DRB1*14:04:01-DRB3*02:02:01	5	-0.03	0.97	0.65	-0.05	9.60E-01
219	DRB1*14:04:01-DRB4*01:01:01:01h	1	>0				
220	DRB1*14:04:01-DRB4*01:03:01h	1	>0				
221	DRB1*14:04:01-nullh	1	<0				
222	DRB1*14:06:01-DRB3*01:01:02	1	<0				
223	DRB1*14:06:01-DRB4*01:03:01h	1	<0				
224	DRB1*14:54:01-DRB3*01:01:02h	1	<0				
225	DRB1*14:54:01-DRB3*02:02:01	6	-0.07	0.93	0.71	-0.09	9.24E-01
226	DRB1*14:54:01-DRB3*03:01:01h	1	>0				
227	DRB1*14:54:01-DRB4*01:03:01h	4	-0.26	0.77	1.00	-0.26	7.96E-01
228	DRB1*15:01:01-DRB3*01:01:02h	1	<0				
229	DRB1*15:01:01-DRB4*01:03:01h	2	>0				
230	DRB1*15:01:01-DRB5*01:01:01	5	>0				
231	DRB1*15:01:01-DRB5*02:02:01h	1	<0				
232	DRB1*15:01:01-nullh	1	<0				

233	DRB1*15:02:01-DRB3*01:01:02h	2	<0				
234	DRB1*15:02:01-DRB4*01:03:01h	5	<0				
236	DRB1*15:02:01-DRB5*01:02	4	<0				
235	DRB1*15:02:01-DRB5*01:02:01	3	<0				
237	DRB1*15:02:02-DRB3*01:01:02h	1					
238	DRB1*15:02:02-DRB5*01:02:01	1					
239	DRB1*16:01:01-DRB3*01:01:02h	6	0.04	1.04	0.71	0.06	9.55E-01
240	DRB1*16:01:01-DRB3*02:02:01h	3	-0.20	0.82	1.00	-0.20	8.41E-01
241	DRB1*16:01:01-DRB3*03:01:01h	1	<0				
242	DRB1*16:01:01-DRB4*01:03:01h	13	-0.50	0.61	0.71	-0.70	4.84E-01
243	DRB1*16:01:01-DRB4*01:03:02h	2	0.31	1.37	0.71	0.44	6.62E-01
244	DRB1*16:01:01-DRB5*01:01:01	2	<0				
245	DRB1*16:01:01-DRB5*02:02:01	25	-0.14	0.87	0.38	-0.36	7.17E-01
246	DRB1*16:02:01-DRB3*02:02:01h	1	<0				
247	DRB1*16:02:01-DRB4*01:03:01h	1	0.64	1.90	1.00	0.64	5.20E-01
248	DRB1*16:02:01-DRB5*02:02:01	1	<0				
249	DRB1*16:02:01-DRB5*02:21	1	0.64	1.90	1.00	0.64	5.20E-01

Table S4. DR-DQ haplotypes, polymorphic motifs of amino acids at (β11, β26) and associated frequencies among 526 carriers of DR3-DQ2.5 with a total number of haplotypes equal to 1052 (=2*526)

ID	DR-DQ Genetic Haplotypes	motif	Freq
1	DRB1*01:01:01-null-DQA1*01:01:01-DQB1*05:01:01	..	24
2	DRB1*01:02:01-null-DQA1*01:01:02-DQB1*05:01:01	..	1
3	DRB1*01:03:01-null-DQA1*01:01:01-DQB1*05:01:01	..	1
4	DRB1*07:01:01-DRB4*01:03:01:02N-DQA1*02:01:01-DQB1*02:02:01	..	1
5	DRB1*07:01:01-DRB4*01:03:01:02N-DQA1*02:01:01-DQB1*03:03:02	..	6
6	DRB1*08:01:01-null-DQA1*04:01:01-DQB1*04:02:01	..	1
7	DRB1*08:02:01-null-DQA1*04:01:01-DQB1*04:02:01	..	1
8	DRB1*08:03:02-null-DQA1*06:01:01-DQB1*03:01:01	..	1
9	DRB1*08:04:01-null-DQA1*04:01:02-DQB1*04:02:13	..	1
10	DRB1*10:01:01-null-DQA1*01:05:01-DQB1*05:01:01	..	3
11	DRB1*04:01:01-DRB4*01:01:01:01-DQA1*03:03:01-DQB1*03:02:01	AN	1
12	DRB1*04:01:01-DRB4*01:01:01-DQA1*03:01:01-DQB1*03:02:01	AN	1
13	DRB1*04:01:01-DRB4*01:01:01-DQA1*03:03:01-DQB1*03:02:01	AN	1
14	DRB1*04:01:01-DRB4*01:03:01-DQA1*03:01:01-DQB1*03:02:01	AN	165
15	DRB1*04:01:01-DRB4*01:03:01-DQA1*03:01:01-DQB1*03:05:03	AN	1
16	DRB1*04:01:01-DRB4*01:03:01-DQA1*03:03:01-DQB1*03:01:01	AN	24
17	DRB1*04:01:01-DRB4*01:03:01-DQA1*03:03:01-DQB1*03:02:01	AN	27
18	DRB1*04:01:01-DRB4*01:03:02-DQA1*03:01:01-DQB1*03:02:01	AN	1
19	DRB1*04:02:01-DRB4*01:03:01-DQA1*03:01:01-DQB1*03:02:01	AN	16
20	DRB1*04:03:01-DRB4*01:03:01-DQA1*03:01:01-DQB1*03:02:01	AN	6
21	DRB1*04:04:01-DRB4*01:03:01-DQA1*03:01:01-DQB1*03:02:01	AN	65
22	DRB1*04:04:01-DRB4*01:03:02-DQA1*03:01:01-DQB1*03:02:01	AN	5
23	DRB1*04:04:01-DRB4*01:03:03-DQA1*03:01:01-DQB1*03:02:01	AN	1
24	DRB1*04:05:01-DRB4*01:03:01-DQA1*03:03:01-DQB1*02:02:01	AN	1
25	DRB1*04:05:01-DRB4*01:03:01-DQA1*03:03:01-DQB1*03:02:01	AN	22

26	DRB1*04:05:01-DRB4*01:03:01-DQA1*03:03:01-DQB1*03:02:02	AN	2
27	DRB1*04:05:01-DRB4*01:03:01-DQA1*03:03:01-DQB1*04:01:01	AN	1
28	DRB1*04:05:01-DRB4*01:03:01-DQA1*05:05:01-DQB1*03:01:01	AN	1
29	DRB1*04:07:01-DRB4*01:03:01-DQA1*03:01:01-DQB1*03:02:01	AN	4
30	DRB1*04:07:01-DRB4*01:03:01-DQA1*03:03:01-DQB1*03:01:01	AN	1
31	DRB1*04:07:01-DRB4*01:03:02-DQA1*03:01:01-DQB1*03:02:01	AN	2
32	DRB1*04:08:01-DRB4*01:03:01-DQA1*03:03:01-DQB1*03:01:01	AN	2
33	DRB1*04:08:01-DRB4*01:03:01-DQA1*03:03:01-DQB1*03:04:01	AN	6
34	DRB1*04:09:01-DRB4*01:03:01-DQA1*03:03:01-DQB1*03:01:01	AN	1
35	DRB1*07:01:01-DRB4*01:01:01:01-DQA1*02:01:01-DQB1*02:02:01	AN	8
36	DRB1*07:01:01-DRB4*01:01:01:01-DQA1*03:03:01-DQB1*02:02:01	AN	1
37	DRB1*07:01:01-DRB4*01:01:01-DQA1*02:01:01-DQB1*02:02:01	AN	5
38	DRB1*07:01:01-DRB4*01:03:01-DQA1*02:01:01-DQB1*02:02:01	AN	5
39	DRB1*07:01:01-DRB4*01:03:01-DQA1*02:01:01-DQB1*03:03:02	AN	1
40	DRB1*09:01:02-DRB4*01:01:01:01-DQA1*03:03:01-DQB1*02:02:01	AN	1
41	DRB1*09:01:02-DRB4*01:03:01-DQA1*03:02:01-DQB1*03:03:02	AN	1
42	DRB1*09:01:02-DRB4*01:03:02-DQA1*03:02:01-DQB1*03:03:02	AN	9
43	DRB1*03:01:01-DRB3*01:02-DQA1*05:01:01-DQB1*02:01:01	CY	2
44	DRB1*15:01:01-DRB5*01:01:01-DQA1*01:02:01-DQB1*06:03:01	DF	1
45	DRB1*15:02:01-DRB5*01:02:01-DQA1*01:03:01-DQB1*06:01:01	DF	1
46	DRB1*15:02:01-DRB5*01:02-DQA1*01:03:01-DQB1*06:01:01	DF	1
47	DRB1*15:02:02-DRB5*01:02:01-DQA1*01:03:01-DQB1*06:01:01	DF	1
48	DRB1*16:01:01-DRB5*02:02:01-DQA1*01:02:02-DQB1*05:02:01	DF	7
49	DRB1*03:01:01-DRB3*02:02:01-DQA1*05:01:01-DQB1*02:01:01	LF	121
50	DRB1*03:01:01-DRB3*02:24-DQA1*05:01:01-DQB1*02:01:01	LF	1
51	DRB1*11:01:01-DRB3*02:02:01-DQA1*05:05:01-DQB1*03:01:01	LF	6
52	DRB1*11:03:01-DRB3*02:02:01-DQA1*05:05:01-DQB1*03:01:01	LF	2
53	DRB1*11:04:01-DRB3*02:02:01-DQA1*05:05:01-DQB1*03:01:01	LF	1
54	DRB1*12:01:01-DRB3*02:02:01-DQA1*05:05:01-DQB1*03:01:01	LF	2
55	DRB1*13:01:01-DRB3*02:02:01-DQA1*01:03:01-DQB1*06:03:01	LF	6
56	DRB1*13:02:01-DRB3*03:01:01-DQA1*01:02:01-DQB1*05:01:01	LF	1

57	DRB1*13:02:01-DRB3*03:01:01-DQA1*01:02:01-DQB1*06:04:01	LF	12
58	DRB1*13:02:01-DRB3*03:01:01-DQA1*01:02:01-DQB1*06:09:01	LF	5
59	DRB1*14:54:01-DRB3*02:02:01-DQA1*01:04:01-DQB1*05:03:01	LF	1
60	DRB1*03:01:01-DRB3*01:01:02-DQA1*05:01:01-DQB1*02:01:01	RY	446
61	DRB1*13:01:01-DRB3*01:01:02-DQA1*01:03:01-DQB1*06:03:01	RY	4
62	DRB1*13:03:01-DRB3*01:01:02-DQA1*05:05:01-DQB1*03:01:01	RY	3

Table S5. DM-DR1 interactions: A) a-chains, a2-a2 domains, pH = 5.5[1], and B) β-chains, β2-β2 domains, pH = 5.5[2]

A) DR residue	DM residue(s)	Type of interaction(s)
100Arg[1]	171Ser/172Asp	Guanidinium—Ser-OH/Asp C=O
100Arg	173Ile/192Val	Hydrophobic interaction of α100R side chains with those of 173I/192V; the latter two also interact mutually
101Glu	194Arg	Carboxyl—guanidinium charge-charge interaction
130Thr	188Ile	Hydrophobic side-chain interactions
131Gly	186Thr	Hydrophobic side-chain interactions
-----	181Glu—184Arg	Intra-chain carboxyl—guanidinium charge-charge interaction
	185Tyr—98Arg	π-cation interaction
	100Phe—98Arg	π-cation interaction
	185Tyr—100Phe	π-π interaction
	125Asn—99Gly	125Asn amide H-bond to C=O of 99Gly

B) DRβ residue(s)	DMβ residue(s)	Type of interaction(s)
98Lys	107Phe	π-cation interaction
99Val	108Asn	Amide group of DMβ108 with carbonyl of DRβ99
184Leu	108Asn	Hydrophobic side-chain interactions
185Thr (C=O)	108Asn (C=O) and 110Arg (NH)	Backbone amide/carbonyl interactions
187Glu	110Arg	Inter-chain carboxyl—guanidinium charge-charge interaction
187Glu—189Arg	(See also previous line on 187Glu)	Intra-chain carboxyl—guanidinium charge-charge interaction
184Leu-99Val-100Thr-185Thr-101Val	(See also third line on 184Leu)	Hydrophobic side chain interactions

[1] Underlined residues show reduced or no activity in DM-mediated DR-CLIP/peptide exchange, when mutated to Ala, as shown by Pos et al., 2012. Pos W, Sethi DK, Call MJ, Schulze MS, Anders AK, Pyrdol J, Wucherpfennig KW. Crystal structure of the HLA-DM-HLA-DR1 complex defines mechanisms for rapid peptide selection. Cell. 2012 Dec 21;151(7):1557-68. doi: 10.1016/j.cell.2012.11.025. PMID: 23260142.

[2] Pos et al., 2012. Pos W, Sethi DK, Call MJ, Schulze MS, Anders AK, Pyrdol J, Wucherpfennig KW. Crystal structure of the HLA-DM-HLA-DR1 complex defines mechanisms for rapid peptide selection. Cell. 2012 Dec 21;151(7):1557-68. doi: 10.1016/j.cell.2012.11.025. PMID: 23260142.

Table S6. Demographic and clinical risk factors in the iCohort, computed frequencies, and association results include coefficient, hazard ratio, standard error, Z-score and p-value, from univariate analysis with one covariate a time (left panel) and multivariate analysis with all covariates (right panel). P-values, if less than 0.05, are highlighted red and green, if corresponding HR is less than 1 or greater than 1, respectively.

			Univariate Analysis					Multivariate Analysis				
**Variable**	**Value**	**n**	**coef**	**HR**	**SE**	**Z**	**p**	**coef**	**HR**	**SE**	**Z**	**p**
**Race**	White	1097	0.00	1.00				0.00	1.00			
	Others	119	-0.03	0.97	0.18	-0.17	8.65E-01	0.07	1.07	0.18	0.37	7.09E-01
**Sex**	Male	708	0.00	1.00				0.00	1.00			
	Female	508	0.12	1.13	0.10	1.21	2.26E-01	0.14	1.16	0.10	1.44	1.50E-01
**Age**	in 10 years	1216	-0.36	0.70	0.01	-4.70	2.65E-06	-0.53	0.59	0.08	-6.33	2.44E-10
**Risk Level**	Low	900	0.00	1.00				0.00	1.00			
	High	316	0.61	1.83	0.08	5.82	5.92E-09	0.87	2.39	0.13	6.49	8.54E-11
**insulin**	Placebo	606	0.00	1.00				0.00	1.00			
	Tretment	610	-0.18	0.83	0.10	1.83	6.76E-02	0.17	1.19	0.10	1.76	7.90E-02
**Study**	DPT1	670	0.00	1.00				0.00	1.00			
	TN07	546	-0.18	0.83	0.10	-1.77	7.71E-02	0.00	1.00	0.13	-0.02	9.84E-01

Table S7. Association results from haplotypic association analysis on HLA-DR haplotypes with the progression: number of haplotypes, coefficient, Hazard ratio, standard error, Z-score and p-value. P-values are highlighted green or yellow for p-value less than 0.05 and corresponding HR is greater (risk) or less (resistant) than 1, respectively. Note that for many uncommon DR haplotypes with fewer than 10 observations, association statistics were not computable, since there were no T1D event(s) observed (coef<0), or there is only one T1D event associated with a single DR haplotype (coef>0).

seq	DR haplotypes	id	n	coef	HR	SE	Z	p
1	DRB1*01:01:01-null	4	125	-0.46	0.63	0.45	-1.02	3.07E-01
2	DRB1*01:02:01-null	20	20	0.26	1.30	0.45	0.59	5.54E-01
3	DRB1*01:03:01-null	25	14	0.90	2.46	0.41	2.22	2.67E-02
4	DRB1*03:01:01-DRB3*01:01:02	2	446	-0.31	0.73	0.15	-2.06	3.94E-02
5	DRB1*03:01:01-DRB3*01:02	46	2	<0				
6	DRB1*03:01:01-DRB3*02:02:01	5	121	0.37	1.45	0.16	2.38	1.72E-02
7	DRB1*03:01:01-DRB3*02:24	50	1	<0				
8	DRB1*03:02:01-DRB3*01:62:01	51	1	<0				
9	DRB1*03:04:01-DRB3*01:01:02	52	1	<0				
10	DRB1*03:124-DRB3*02:02:01	53	1	<0				
11	DRB1*04:01:01-DRB4*01:01:01	34	5	-0.80	0.45	1.01	-0.80	4.24E-01
12	DRB1*04:01:01-DRB4*01:01:01:01	54	1	0.82	2.27	1.03	0.80	4.26E-01
13	DRB1*04:01:01-DRB4*01:03:01	1	740	0.03	1.03	0.11	0.24	8.13E-01
14	DRB1*04:01:01-DRB4*01:03:02	39	4	-0.54	0.58	1.01	-0.54	5.91E-01
15	DRB1*04:01:03-DRB4*01:03:01	55	1	<0				
16	DRB1*04:02:01-DRB4*01:03:01	8	54	0.18	1.20	0.20	0.91	3.64E-01
17	DRB1*04:03:01-DRB4*01:03:01	26	12	0.17	1.19	0.46	0.38	7.06E-01
18	DRB1*04:04:01-DRB4*01:03:01	3	145	-0.16	0.85	0.16	-1.00	3.18E-01
19	DRB1*04:04:01-DRB4*01:03:02	19	21	0.06	1.06	0.36	0.16	8.70E-01
20	DRB1*04:04:01-DRB4*01:03:03	56	1	0.49	1.63	1.01	0.48	6.29E-01
21	DRB1*04:05:01-DRB4*01:03:01	7	70	0.07	1.08	0.26	0.28	7.79E-01
22	DRB1*04:07:01-DRB4*01:03:01	21	20	-0.44	0.65	0.58	-0.75	4.52E-01
23	DRB1*04:07:01-DRB4*01:03:02	28	8	-0.16	0.85	0.72	-0.23	8.19E-01
24	DRB1*04:08:01-DRB4*01:03:01	15	25	0.15	1.17	0.36	0.42	6.72E-01
25	DRB1*04:08:01-DRB4*01:03:02	57	1	>0				

26	DRB1*04:09:01-DRB4*01:03:01	58	1	<0				
27	DRB1*04:10:01-DRB4*01:03:02	59	1	<0				
28	DRB1*04:11:01-DRB4*01:03:01	42	3	<0				
29	DRB1*04:13-DRB4*01:03:01	60	1	<0				
30	DRB1*07:01:01-DRB4*01:01:01	9	48	0.52	1.68	0.33	1.59	1.12E-01
31	DRB1*07:01:01-DRB4*01:01:01:01	17	24	-0.32	0.73	0.45	-0.70	4.82E-01
32	DRB1*07:01:01-DRB4*01:03:01	11	44	-0.40	0.67	0.37	-1.07	2.83E-01
33	DRB1*07:01:01-DRB4*01:03:01:02N	27	12	-0.36	0.70	0.72	-0.50	6.15E-01
34	DRB1*07:01:01-DRB4*01:06	61	1	>0				
35	DRB1*08:01:01-null	10	46	0.27	1.30	0.26	1.02	3.06E-01
36	DRB1*08:02:01-null	29	8	<0				
37	DRB1*08:03:02-null	43	3	<0				
38	DRB1*08:04:01-null	35	5	0.37	1.44	1.01	0.36	7.16E-01
39	DRB1*08:11:01-null	62	1	<0				
40	DRB1*09:01:02-DRB4*01:01:01	31	6	-0.28	0.76	0.72	-0.38	7.04E-01
41	DRB1*09:01:02-DRB4*01:01:01:01	47	2	<0				
42	DRB1*09:01:02-DRB4*01:03:01	48	2	<0				
43	DRB1*09:01:02-DRB4*01:03:02	18	22	0.33	1.40	0.36	0.92	3.56E-01
44	DRB1*10:01:01-null	30	8	<0				
45	DRB1*11:01:01-DRB3*02:02:01	13	41	-0.73	0.48	0.39	-1.90	5.78E-02
46	DRB1*11:01:01-DRB3*02:167	63	1	1.72	5.59	1.03	1.67	9.52E-02
47	DRB1*11:01:02-DRB3*02:02:01	44	3	1.00	2.72	0.73	1.38	1.68E-01
48	DRB1*11:02:01-DRB3*02:02:01	36	5	1.21	3.36	0.62	1.97	4.88E-02
49	DRB1*11:03:01-DRB3*02:02:01	32	6	-0.57	0.56	1.03	-0.56	5.77E-01
50	DRB1*11:04:01-DRB3*02:02:01	23	16	-0.35	0.71	0.62	-0.56	5.77E-01
51	DRB1*11:12:01-DRB3*02:02:01	64	1	1.15	3.16	1.03	1.12	2.65E-01
52	DRB1*12:01:01-DRB3*02:02:01	22	19	0.77	2.17	0.40	1.95	5.09E-02
53	DRB1*12:02:01-DRB3*03:01:03	65	1	<0				
54	DRB1*13:01:01-DRB3*01:01:02	12	44	0.22	1.24	0.42	0.52	6.06E-01
55	DRB1*13:01:01-DRB3*02:02:01	14	32	-0.34	0.71	0.43	-0.78	4.37E-01
56	DRB1*13:02:01-DRB3*03:01:01	6	103	-0.29	0.75	0.44	-0.64	5.20E-01
57	DRB1*13:03:01-DRB3*01:01:02	24	16	0.16	1.17	0.53	0.30	7.64E-01

58	DRB1*13:04-DRB3*03:01:01	66	1	<0				
59	DRB1*13:05:01-DRB3*02:02:01	37	5	-0.73	0.48	1.03	-0.70	4.81E-01
60	DRB1*13:20:01-DRB3*01:01:02	67	1	<0				
61	DRB1*14:02:01-DRB3*01:01:02	68	1	<0				
62	DRB1*14:02:01-DRB3*02:02:01	49	2	1.10	3.01	1.04	1.06	2.91E-01
63	DRB1*14:04:01-DRB3*02:02:01	40	4	0.06	1.07	0.72	0.09	9.29E-01
64	DRB1*14:06:01-DRB3*01:01:02	69	1	<0				
65	DRB1*14:54:01-DRB3*02:02:01	33	6	-0.07	0.93	0.72	-0.10	9.23E-01
66	DRB1*15:01:01-DRB5*01:01:01	38	5	1.33	3.79	1.01	1.32	1.88E-01
67	DRB1*15:02:01-DRB5*01:02	41	4	<0				
68	DRB1*15:02:01-DRB5*01:02:01	45	3	<0				
69	DRB1*15:02:02-DRB5*01:02:01	70	1					
70	DRB1*16:01:01-DRB5*01:01:01	71	1	<0				
71	DRB1*16:01:01-DRB5*02:02:01	16	25	-0.16	0.86	0.40	-0.39	7.00E-01
72	DRB1*16:02:01-DRB5*02:02:01	72	1	<0				
73	DRB1*16:02:01-DRB5*02:21	73	1	0.65	1.92	1.01	0.65	5.18E-01

Table S8. Association results from assessing DR somatic haplotypes (four copies per individual) association with the progression from stage 1/2 disease to onset of clinical type 1 diabetes (stage 3) with (right panel) and without (left panel) adjusting for HLA-DQ genotypes: number of haplotypes, coefficient, Hazard ratio, standard error, Z-score and p-value. P-values are highlighted green or yellow for p-value less than 0.05 and corresponding HR is greater (risk) or less (resistant) than 1, respectively. Note that suffix "h" indicates somatic DR haplotype not observed in the population.

seq	DRB1-DRB3/4/5[1]	n	Without Adjusting HLA-DQ					Adjusting HLA-DQ				
			coef	HR	SE	Z	p	coef	HR	SE	Z	p
1	*01:01:01-DRB3*01:01:02h	23	0.48	1.61	0.31	1.55	1.20E-01	0.76	2.14	0.35	2.19	2.85E-02
2	*01:01:01-DRB4*01:03:01h	64	-0.06	0.94	0.22	-0.27	7.86E-01	-0.36	0.70	0.43	-0.83	4.09E-01
3	*01:01:01-null	145	-0.14	0.87	0.12	-1.19	2.33E-01	-1.05	0.35	0.38	-2.79	5.28E-03
4	*01:02:01-DRB4*01:03:01h	11	0.11	1.11	0.51	0.21	8.33E-01	0.63	1.88	0.66	0.95	3.41E-01
5	*01:02:01-null	27	0.00	1.00	0.27	0.00	9.97E-01	0.17	1.18	0.27	0.63	5.31E-01
6	*01:03:01-null	17	0.27	1.31	0.29	0.92	3.56E-01	0.54	1.72	0.31	1.77	7.72E-02
7	*03:01:01-DRB3*01:01:02	526	0.02	1.02	0.07	0.38	7.06E-01	-0.32	0.73	0.13	-2.50	1.23E-02
8	*03:01:01-DRB3*02:02:01	154	0.31	1.37	0.10	3.01	2.59E-03	0.30	1.35	0.12	2.43	1.51E-02
9	*03:01:01-DRB3*03:01:01h	18	-0.74	0.48	0.58	-1.27	2.05E-01	-0.51	0.60	0.60	-0.85	3.95E-01
10	*03:01:01-DRB4*01:01:01:01h	11	-0.80	0.45	0.71	-1.13	2.60E-01	-0.81	0.45	0.73	-1.11	2.67E-01
11	*03:01:01-DRB4*01:03:01h	352	0.24	1.27	0.10	2.26	2.38E-02	-0.22	0.80	0.35	-0.63	5.31E-01
12	*03:01:01-DRB4*01:03:02h	17	0.38	1.46	0.38	1.00	3.18E-01	0.35	1.42	0.39	0.91	3.61E-01
13	*03:01:01h[1]	33	0.23	1.25	0.27	0.83	4.08E-01	0.45	1.57	0.31	1.47	1.41E-01
14	*04:01:01-DRB3*01:01:02h	201	0.05	1.05	0.13	0.39	6.98E-01	-0.21	0.81	0.16	-1.30	1.92E-01
15	*04:01:01-DRB3*02:02:01h	100	0.08	1.09	0.17	0.49	6.23E-01	0.07	1.07	0.18	0.39	6.99E-01
16	*04:01:01-DRB3*03:01:01h	41	-0.54	0.58	0.32	-1.69	9.10E-02	-0.21	0.81	0.36	-0.57	5.71E-01
17	*04:01:01-DRB4*01:01:01	31	-0.03	0.97	0.27	-0.11	9.13E-01	0.00	1.00	0.29	0.01	9.94E-01
18	*04:01:01-DRB4*01:03:01	997	0.02	1.02	0.05	0.51	6.13E-01	0.02	1.02	0.07	0.33	7.39E-01
19	*04:01:01-DRB4*01:03:02	18	0.41	1.51	0.32	1.29	1.98E-01	0.42	1.52	0.34	1.25	2.11E-01
20	*04:01:01-DRB5*02:02:01h	11	-0.40	0.67	0.58	-0.70	4.86E-01	-0.19	0.83	0.59	-0.32	7.47E-01
21	*04:01:01h	88	-0.04	0.96	0.19	-0.20	8.42E-01	0.10	1.10	0.24	0.40	6.92E-01
22	*04:02:01-DRB3*01:01:02h	10	0.74	2.10	0.45	1.65	9.91E-02	0.63	1.88	0.46	1.38	1.69E-01

23	*04:02:01-DRB3*02:02:01h	11	0.42	1.53	0.38	1.11	2.69E-01	0.25	1.28	0.39	0.63	5.28E-01
24	*04:02:01-DRB4*01:03:01	75	0.13	1.14	0.12	1.08	2.80E-01	0.05	1.05	0.13	0.39	6.94E-01
25	*04:03:01-DRB4*01:03:01	16	0.15	1.16	0.33	0.45	6.53E-01	0.01	1.01	0.33	0.03	9.80E-01
26	*04:04:01-DRB3*01:01:02h	61	0.12	1.12	0.21	0.57	5.70E-01	-0.11	0.89	0.23	-0.50	6.18E-01
27	*04:04:01-DRB3*02:02:01h	33	0.34	1.40	0.28	1.19	2.32E-01	0.20	1.22	0.32	0.63	5.30E-01
28	*04:04:01-DRB4*01:03:01	192	0.02	1.02	0.11	0.22	8.26E-01	-0.12	0.89	0.12	-1.01	3.15E-01
29	*04:04:01-DRB4*01:03:02	25	0.16	1.18	0.31	0.51	6.08E-01	0.08	1.08	0.32	0.24	8.14E-01
30	*04:04:01h	11	-0.57	0.56	0.58	-0.99	3.23E-01	-0.85	0.43	0.62	-1.37	1.71E-01
31	*04:05:01-DRB3*01:01:02h	21	0.29	1.34	0.38	0.76	4.46E-01	0.19	1.21	0.42	0.46	6.45E-01
32	*04:05:01-DRB3*02:02:01h	15	0.05	1.05	0.50	0.10	9.24E-01	-0.15	0.86	0.53	-0.27	7.85E-01
33	*04:05:01-DRB4*01:03:01	88	0.12	1.13	0.14	0.85	3.98E-01	0.05	1.05	0.18	0.28	7.77E-01
34	*04:07:01-DRB4*01:03:01	31	-0.55	0.58	0.42	-1.32	1.89E-01	-0.53	0.59	0.42	-1.26	2.08E-01
35	*04:08:01-DRB3*01:01:02h	10	0.30	1.34	0.45	0.65	5.14E-01	0.55	1.74	0.55	0.99	3.20E-01
36	*04:08:01-DRB4*01:03:01	36	0.02	1.02	0.23	0.07	9.48E-01	0.13	1.14	0.24	0.56	5.75E-01
37	*07:01:01-DRB3*01:01:02h	28	-0.07	0.93	0.36	-0.19	8.48E-01	0.04	1.04	0.38	0.11	9.11E-01
38	*07:01:01-DRB3*02:02:01h	12	-0.14	0.87	0.58	-0.24	8.13E-01	0.11	1.11	0.61	0.17	8.62E-01
39	*07:01:01-DRB4*01:01:01:01	25	-0.47	0.63	0.41	-1.13	2.59E-01	-0.32	0.73	0.45	-0.71	4.77E-01
40	*07:01:01-DRB4*01:01:01	52	0.17	1.19	0.24	0.72	4.71E-01	0.49	1.64	0.33	1.49	1.36E-01
41	*07:01:01-DRB4*01:03:01:02N	12	-0.50	0.61	0.71	-0.71	4.79E-01	-0.36	0.70	0.72	-0.50	6.15E-01
42	*07:01:01-DRB4*01:03:01	98	-0.24	0.78	0.16	-1.49	1.37E-01	-0.39	0.68	0.24	-1.62	1.06E-01
43	*07:01:01h	18	0.71	2.04	0.34	2.11	3.51E-02	1.05	2.85	0.37	2.79	5.29E-03
44	*08:01:01-DRB4*01:03:01h	28	0.10	1.11	0.31	0.33	7.38E-01	0.23	1.26	0.32	0.73	4.65E-01
45	*08:01:01-null	53	0.09	1.09	0.20	0.43	6.68E-01	0.26	1.29	0.21	1.21	2.26E-01
46	*09:01:02-DRB4*01:03:01	11	-0.13	0.88	0.50	-0.26	7.96E-01	-0.04	0.96	0.51	-0.09	9.30E-01
47	*09:01:02-DRB4*01:03:02	24	0.10	1.11	0.30	0.35	7.30E-01	0.31	1.36	0.31	0.98	3.25E-01
48	*10:01:01-null	10										
49	*11:01:01-DRB3*02:02:01	47	-0.45	0.64	0.27	-1.70	8.86E-02	-0.48	0.62	0.33	-1.44	1.49E-01
50	*11:01:01-DRB4*01:03:01h	27	-0.31	0.73	0.36	-0.87	3.87E-01	-0.50	0.60	0.44	-1.15	2.51E-01
51	*11:04:01-DRB3*02:02:01	19	-0.50	0.61	0.49	-1.01	3.13E-01	-0.42	0.66	0.56	-0.75	4.54E-01
52	*11:04:01-DRB4*01:03:01h	10	0.10	1.11	0.58	0.18	8.57E-01	0.00	1.00	0.63	0.00	9.99E-01
53	*12:01:01-DRB3*02:02:01	19	0.46	1.59	0.36	1.30	1.94E-01	0.77	2.17	0.40	1.95	5.09E-02

54	*12:01:01-DRB4*01:03:01h	14	0.28	1.33	0.45	0.62	5.33E-01	0.47	1.61	0.49	0.97	3.33E-01
55	*13:01:01-DRB3*01:01:02	54	-0.16	0.85	0.27	-0.60	5.50E-01	-0.10	0.91	0.37	-0.26	7.93E-01
56	*13:01:01-DRB3*02:02:01	36	-0.22	0.80	0.28	-0.80	4.24E-01	-0.15	0.86	0.37	-0.42	6.76E-01
57	*13:01:01-DRB4*01:03:01h	51	-0.16	0.85	0.27	-0.59	5.53E-01	-0.07	0.93	0.46	-0.15	8.81E-01
58	*13:02:01-DRB3*01:01:02h	15	-1.13	0.32	0.71	-1.59	1.13E-01	-0.89	0.41	0.72	-1.23	2.18E-01
59	*13:02:01-DRB3*03:01:01	109	-0.46	0.63	0.20	-2.32	2.05E-02	-0.92	0.40	0.72	-1.28	2.02E-01
60	*13:02:01-DRB4*01:03:01h	56	-0.30	0.74	0.26	-1.18	2.38E-01	0.13	1.14	0.33	0.39	6.94E-01
61	*13:03:01-DRB3*01:01:02	19	-0.26	0.77	0.42	-0.62	5.33E-01	-0.07	0.93	0.44	-0.17	8.65E-01
62	*16:01:01-DRB4*01:03:01h	13	-0.74	0.48	0.71	-1.05	2.96E-01	-0.52	0.60	0.72	-0.72	4.74E-01
63	*16:01:01-DRB5*02:02:01	25	-0.37	0.69	0.38	-0.96	3.35E-01	-0.16	0.86	0.40	-0.39	7.00E-01
	uncommon haplotypes (<10)	451	-0.09	0.92	0.08	-1.13	2.60E-01	0.02	1.02	0.09	0.26	7.91E-01

[1] indicates a somatic haplotype of DRB1 allele without a DRB3/4/5 allele

REFERENCES

1. Petersen J, Llerena C, Golzarroshan B, Faoro C, Triebel F, Rossjohn J. Crystal structure of the human LAG-3-HLA-DR1-peptide complex. Sci Immunol. 2024 Dec 13;9(102):eads5122. doi: 10.1126/sciimmunol.ads5122. Epub 2024 Dec 13. PMID: 39671469.

2. Wang N, Waghray D, Caveney NA, Jude KM, Garcia KC. Structural insights into human MHC-II association with invariant chain. Proc Natl Acad Sci U S A. 2024 May 7;121(19):e2403031121. doi: 10.1073/pnas.2403031121. Epub 2024 Apr 30. PMID: 38687785.
